# The *Candida albicans* quorum-sensing molecule farnesol alters sphingolipid metabolism in human monocyte-derived dendritic cells

**DOI:** 10.1128/mbio.00732-24

**Published:** 2024-07-02

**Authors:** Maria Batliner, Fabian Schumacher, Dominik Wigger, Wolfgang Vivas, Agata Prell, Ingo Fohmann, Tobias Köhler, Rebekka Schempp, Angela Riedel, Martin Vaeth, Agnes Fekete, Burkhard Kleuser, Oliver Kurzai, Natalie E. Nieuwenhuizen

**Affiliations:** 1 Institute for Hygiene and Microbiology, Julius-Maximilians University of Würzburg, Würzburg, Germany; 2 Institute of Pharmacy, Freie Universität Berlin, Berlin, Germany; 3 Institute for Infectious Diseases and Infection Control, Jena University Hospital–Friedrich Schiller University, Jena, Germany; 4 Associated Research Group Translational Infection Medicine, Leibniz Institute for Natural Product Research and Infection Biology–Hans Knoell Institute (HKI), Jena, Germany; 5 Department of Anesthesiology and Intensive Care Medicine, Jena University Hospital–Friedrich Schiller University, Jena, Germany; 6 Institute for Virology and Immunobiology, Julius-Maximilians University of Würzburg, Würzburg, Germany; 7 Mildred Scheel Early Career Center (MSNZ), University Hospital of Würzburg, Würzburg, Germany; 8 Max Planck Research Group, Würzburg Institute of Systems Immunology, Julius-Maximilians University of Würzburg, Würzburg, Germany; 9 Pharmaceutical Biology, Julius-von-Sachs-Institute, Biocenter, University of Würzburg, Würzburg, Germany; 10 Research Group Fungal Septomics, Leibniz Institute for Natural Product Research and Infection Biology–Hans Knoell Institute, Jena, Germany; 11 National Reference Center for Invasive Fungal Infections, Leibniz Institute for Natural Product Research and Infection Biology–Hans Knoell Institute, Jena, Germany; Hebrew University of Jerusalem Robert H. Smith Faculty of Agriculture Food and Environment, Rehovot, Israel

**Keywords:** farnesol, *Candida albicans*, sphingolipids, dihydroceramide, oxidative stress, mitochondrial metabolism, fungi, serine palmitoyltransferase, dihydroceramide desaturase, quorum-sensing, dendritic cells, monocytes

## Abstract

*Candida albicans,* an opportunistic fungal pathogen, produces the quorum-sensing molecule farnesol, which we have shown alters the transcriptional response and phenotype of human monocyte-derived dendritic cells (DCs), including their cytokine secretion and ability to prime T cells. This is partially dependent on the nuclear receptor peroxisome proliferator-activated receptor gamma (PPAR-γ), which has numerous ligands, including the sphingolipid metabolite sphingosine 1-phosphate. Sphingolipids are a vital component of membranes that affect membrane protein arrangement and phagocytosis of *C. albicans* by DCs. Thus, we quantified sphingolipid metabolites in monocytes differentiating into DCs by High-performance liquid chromatography-tandem mass spectrometry (HPLC-MS/MS). Farnesol increased the activity of serine palmitoyltransferase, leading to increased levels of 3-keto-dihydrosphingosine, dihydrosphingosine, and dihydrosphingosine 1-phosphate and inhibited dihydroceramide desaturase by inducing oxidative stress, leading to increased levels of dihydroceramide and dihydrosphingomyelin species and reduced ceramide levels. Accumulation of dihydroceramides can inhibit mitochondrial function; accordingly, farnesol reduced mitochondrial respiration. Dihydroceramide desaturase inhibition increases lipid droplet formation, which we observed in farnesol-treated cells, coupled with an increase in intracellular triacylglycerol species. Furthermore, inhibition of dihydroceramide desaturase with either farnesol or specific inhibitors impaired the ability of DCs to prime interferon-γ-producing T cells. The effect of farnesol on sphingolipid metabolism, triacylglycerol synthesis, and mitochondrial respiration was not dependent on PPAR-γ. In summary, our data reveal novel effects of farnesol on sphingolipid metabolism, neutral lipid synthesis, and mitochondrial function in DCs that affect their instruction of T cell cytokine secretion, indicating that *C. albicans* can manipulate host cell metabolism via farnesol secretion.

**IMPORTANCE:**

*Candida albicans* is a common commensal yeast, but it is also an opportunistic pathogen which is one of the leading causes of potentially lethal hospital-acquired infections. There is growing evidence that its overgrowth in the gut can influence diseases as diverse as alcohol-associated liver disease and COVID-19. Previously, we found that its quorum-sensing molecule, farnesol, alters the phenotype of dendritic cells differentiating from monocytes, impairing their ability to drive protective T cell responses. Here, we demonstrate that farnesol alters the metabolism of sphingolipids, important structural components of the membrane that also act as signaling molecules. In monocytes differentiating to dendritic cells, farnesol inhibited dihydroceramide desaturase, resulting in the accumulation of dihydroceramides and a reduction in ceramide levels. Farnesol impaired mitochondrial respiration, known to occur with an accumulation of dihydroceramides, and induced the accumulation of triacylglycerol and oil bodies. Inhibition of dihydroceramide desaturase resulted in the impaired ability of DCs to induce interferon-γ production by T cells. Thus, farnesol production by *C. albicans* could manipulate the function of dendritic cells by altering the sphingolipidome.

## INTRODUCTION

The yeast *Candida albicans* is a common human commensal that can cause opportunistic superficial infections in immunocompromised individuals and invasive infections with high mortality rates in the hospital setting (1). There is also growing evidence that the overgrowth of *C. albicans* in the gut can affect diseases as diverse as alcoholic liver disease and COVID-19 (2, 3). *C. albicans* infections are often characterized by the formation of biofilms, in which the ability of the fungus to switch between yeast and hyphal form plays an important role (4). *C. albicans* secretes the quorum-sensing molecule farnesol (FOH), an acyclic sesquiterpene alcohol, in a cell density-dependent manner to regulate filamentous growth and biofilm formation (5, 6). This lipophilic molecule is generated by the dephosphorylation of farnesyl pyrophosphate as a byproduct of the mevalonate pathway (7). The levels of FOH secreted by *C. albicans* are influenced by temperature and nutrient conditions and can vary strongly depending on the growth cycle and cell density, with yeast cells secreting around 2 µM in the stationary phase and up to 55 µM during active growth (8–10). It is expected that even higher concentrations are reached in biofilms (10).

Neutrophils and monocytes are an important first line of defense against fungal infections, while dendritic cells (DCs) link innate and adaptive immunity via antigen presentation (11, 12). In a previous study, we found that FOH activates neutrophils and monocytes, promoting the oxidative burst and release of inflammatory cytokines, but it did not enhance fungal uptake or killing (13). In addition, in the presence of FOH, DCs derived from primary human monocytes showed impaired expression of several antigen presentation and maturation markers, a reduced ability to prime T cells, and decreased production of IL-12. Interestingly, the lipid antigen-presenting molecule CD1d was upregulated in FOH-treated monocyte-derived DCs, while CD1a was downregulated (13). Upregulation of CD1d was dependent on the activation of the nuclear receptor peroxisome proliferator-activated receptor γ (PPAR-γ) and retinoic acid receptor alpha (RARα) (14). PPAR-γ is a transcription factor that is crucial for energy homeostasis and adipogenesis (15, 16) and is activated by the binding of small lipophilic ligands, including fatty acids, retinoic acid, and the bioactive sphingolipid sphingosine 1-phosphate (S1P) (16, 17). A study found strikingly similar effects of S1P on DC differentiation, with S1P decreasing CD1a expression and IL-12 secretion and increasing IL-10 secretion (18), but this study did not investigate CD1d expression. Thus, we hypothesized that the effects of FOH may be connected to S1P and aimed to investigate the effect of FOH on sphingolipid metabolism.

Sphingolipids are a class of lipids with a sphingoid base backbone, which play an important structural role as components of membranes and include bioactive metabolites that control various cellular functions such as immunity and inflammation (19). The *de novo* synthesis of sphingolipids occurs at the outer leaflet of the endoplasmic reticulum (ER), where serine palmitoyltransferase (SPT) catalyzes the condensation of palmitoyl-CoA and l-serine to form 3-ketodihydrosphingosine (3KDS), which is subsequently converted into downstream sphingolipid metabolites by additional ER-bound enzymes (20). Sphingolipids regulate immune responses to a wide range of bacterial and fungal pathogens, including *C. albicans* (21, 22). Phagocytosis of fungal pathogens relies heavily on the expression and distribution of cell surface receptors such as pattern recognition, Fc-γ, and complement receptors, which are influenced by the membrane lipid composition (23). Thus, the disruption of sphingolipid biosynthesis in DCs highly impaired phagocytosis of *C. albicans*, associated with reduced surface expression of dectin-1, toll-like receptor 2, and Fc-γ receptor (24).

Here, we analyzed the effect of FOH on the sphingolipid composition during monocyte differentiation to DCs. FOH activated SPT, the first enzyme in the *de novo* sphingolipid synthesis pathway, and inhibited dihydroceramide desaturase (Des), the fourth and last enzyme in the *de novo* synthesis cascade, by increasing oxidative stress. Together, this led to an accumulation of dihydroceramides (dhCer), associated with impaired mitochondrial respiration and an increase in oil bodies. Furthermore, the inhibition of Des was associated with a decreased ability of the DCs to secrete IFN-β or induce interferon-γ (IFN-γ) production by T cells. Thus, *C. albicans* can modulate DC metabolism and function by modulating the sphingolipid metabolism via FOH secretion.

## MATERIALS AND METHODS

Additional details are given in the supplementary material.

### Generation of monocyte-derived dendritic cells

Primary human monocytes were isolated from leukocyte reduction system chambers using CD14 MicroBeads (Miltenyi Biotec). CD14^+^ monocytes were seeded in 6-well cell culture plates (2 × 10^6^ monocytes/3 mL medium/well) cultured in RPMI 1640 medium supplemented with 10% heat-inactivated FBS, 10 mM l-glutamine, and 100 U/mL penicillin-streptomycin (PenStrep), and differentiation was induced using IL-4 (1,000 U/mL; Miltenyi Biotec) and GM-CSF [800 U/mL; Leukine (sargramostim)]. Monocytes were treated with FOH (50 or 100 µM; Sigma-Aldrich) or methanol (<0.01%) as a solvent control directly after cell seeding or were left untreated. Some experiments required a 1 h pre-incubation of PPAR-γ antagonist GW9662 (10 µM; Sigma-Aldrich) prior to FOH stimulation. When indicated, monocytes were differentiated in the presence of the PPAR-γ agonist rosiglitazone (RSG; 5 µM; Sigma-Aldrich). Additionally, some monocytes were differentiated on top of a *C. albicans* biofilm (SC5314 or ATCC10231) using transwell inserts (0.4 µM pore size, Corning Costar). Differentiation into immature DCs took place over 6 days (144 h) at 37°C with 5% CO_2_, as previously described (13, 14). Medium exchange and supplementation with cytokines (IL-4, 500 U/mL; GM-CSF, 800 U/mL) and FOH, GW9662, RSG, or solvent control stimulation were carried out on day 3. When indicated, the cells were treated with fenretinide (4-HPR; 5 µM; Sigma-Aldrich) or resveratrol (RV; 50 µM; Cayman Chemical) on day 5 for 24 h. Cells were harvested at different time points to detect changes during the differentiation process. Immature DCs were stimulated on day 6 with lipopolysaccharides (LPS; 100 ng/mL; InvivoGen) for 24 h to induce maturation. Each experiment was conducted with cells from at least three independent donors.

### Characterization of DCs

CD1a and CD1d expressions were determined by flow cytometry. Secretion of IL-1β and IFN-β was measured using DuoSet ELISA kits according to the manufacturer’s protocol (R&D systems). NADPH/NADP+ and glutathione (GSH/GSSG) content were quantified using NADP/NADPH-Glo Assay and GSH/GSSG-Glo Assay kits according to the manufacturer’s protocol (both Promega), respectively. Lipid droplet formation was measured using 4,4-difluoro-1,3,5,7,8-pentamethyl-4-bora-3a,4a-diaza-s-indacene (BODIPY 493/503; 1 µM for 15 min at 37°C with 5% CO_2_; Thermo Fisher) by flow cytometry. Fatty acid uptake was measured using 4,4-difluoro-5,7-dimethyl-4-bora-3a,4a-diaza-s-indacene-3-dodecanoic acid (BODIPY FL C_12_). DCs were serum-starved for 1 h prior to staining with BODIPY FL C_12_ (1 µM for 10 min at 37°C with 5% CO_2_; Thermo Fisher) for uptake analysis by flow cytometry.

### Quantification of cellular sphingolipids by HPLC-MS/MS

Lipids were extracted from cells using 1.5 mL methanol/chloroform (2:1, vol/vol) as previously described (25). The extraction solvent contained d_7_-sphingosine (d_7_-Sph), d_7_-dihydrosphingosine (d_7_-dhSph), d_7_-sphingosine 1-phosphate (d_7_-S1P), C17:0 ceramide (C17:0 Cer), and d_31_-C16:0 sphingomyelin (d_31_-C16:0 SM) (all Avanti Polar Lipids) as internal standards. Chromatographic separations were achieved on a 1290 Infinity II HPLC (Agilent Technologies) equipped with a Poroshell 120 EC-C8 column (3.0 × 150 mm, 2.7 µm; Agilent Technologies). MS/MS analyses were carried out using a 6495C triple-quadrupole mass spectrometer (Agilent Technologies) operating in the positive electrospray ionization mode. Chromatographic conditions and ion source settings of the MS/MS detector have been described elsewhere (26). Sphingolipid subspecies were quantified by multiple reaction monitoring using the mass transitions presented in Table S1. Peak areas of sphingolipid subspecies [dhCer, ceramide (Cer), dihydrosphingomyelin (dhSM), sphingomyelin (SM), and 1-deoxy-sphingolipids], as determined with MassHunter Quantitative Analysis software (version 10.1, Agilent Technologies), were normalized to those of their corresponding internal standards followed by external calibration. Sph, dhSph, and S1P were directly quantified via their deuterated internal standards d_7_-Sph (0.25 pmol on column), d_7_-dhSph, and d_7_-S1P (each 0.125 pmol on column). Dihydrosphingosine 1-phosphate (dhS1P) was quantified via d_7_-S1P. Determined sphingolipid amounts were normalized to cell count.

### Quantification of cellular neutral lipids by UPLC-qTOF-MS

Cellular neutral lipids were extracted from DCs during the differentiation process using the Bligh-Dyer method (27). To detect sphingolipid-associated changes in triacylglycerol (TAG) synthesis, differentiated DCs (144 h) were fed with and without d_7_-dhSph (1 µM for 4 h) prior to lipid extraction. Briefly, cell pellets stored frozen at −80°C were homogenized in methanol, chloroform containing the internal standard TAG 30:0 (100 ng/sample), and water at a ratio of 2:3:1 using ceramic beads. Lipid analysis was performed on an ACQUITY UPLC system coupled to a Synapt G2 HDMS qTOF-MS (all Waters) according to a previously published protocol (28). Acquiring and processing of chromatograms, peak detection, and integration were performed using MassLynx, MarkerLynx, and QuanLynx (version 4.1; all Waters).

### Quantification of cellular free total fatty acids by UPLC-qTOF-MS

To quantify cellular total fatty acids, frozen DC (144 h) pellets were homogenized in 500 µL of isopropanol containing tripotassium phosphate and 10% potassium hydroxide using ceramic beads. Hydrolysis was achieved by heating the mixture at 60°C for 1 h. The pH was then adjusted to ~6 by adding formic acid. After centrifugation (14,000 rpm, 10 min), the supernatants were analyzed by ultra-high performance liquid chromatography-quadrupole time-of-flight mass spectrometry (UPLC-qTOF-MS) using the conditions described in a previous publication (28).

### Cell-free sphingolipid *de novo* synthesis assay

The effects of FOH and retinoic acid (ROH; Sigma-Aldrich) on the sphingolipid *de novo* synthesis were investigated with a microsomal assay that we recently established (20), using human liver microsomes.

### Determination of intracellular Des activity by HPLC-MS/MS

Primary human monocytes were differentiated in the presence of FOH (50 and 100 µM), methanol, or left untreated for 24 h [with and without *N*-acetyl-l-cysteine (NAC) (Sigma-Aldrich)], 72, and 144 h, as described above. Some cells were treated with GW9662 prior to FOH treatment or with RSG for 24 and 72 h. Suspensions of cells were incubated for 4 h with 1 µM of the stable isotope-labeled Des substrate d_7_-C13:0 dhCer (Avanti Polar Lipids) and were subsequently subjected to lipid extraction. The extraction solvent solely contained C17:0 Cer as an internal standard. A 1290 Infinity II HPLC coupled to a 6465B (Ultivo) triple-quadrupole mass spectrometer (both Agilent Technologies) was used for analysis, and instrumental parameters were maintained compared to the sphingolipid profiling described above. Intracellular Des activity was determined as conversion rate (%) = *c* (d_7_-C13:0 Cer)/[*c* (d_7_-C13:0 dhCer) + *c* (d_7_-C13:0 Cer)] with *c* being the concentration (in nmol/L) in the lipid extract.

### Generation of reactive oxygen species

The generation of reactive oxygen species (ROS) was measured using 2′,7′-dichlorofluorescein diacetate (H2DCFDA; 10 µM; Sigma-Aldrich). The following ROS inhibitors were administered for 1 h prior to FOH treatment (4 and 8 h): diphenyliodonium chloride (DPI; 10 µM; Sigma-Aldrich), GSK2795039 (GSK; 50 µM; Cayman Chemical), rotenone (1 and 10 µM; AdipoGen Life Sciences), antimycin A (1 and 10 µM; Sigma-Aldrich), myxothiazol (10 µM; Sigma-Aldrich), S3QEL 2 (10 µM; Sigma-Aldrich), or oligomycin complex (1 and 10 µM; Cayman Chemical). Alternatively, cells were treated with the antioxidant NAC (10 mM; Sigma-Aldrich) together with FOH (100 µM) for 4 h. Additionally, ROS was detected in cells treated with RSG (5 µM; 4 and 8 h) and in cells pre-treated with GW9662 (10 µM for 1 h) followed by FOH stimulation (100 µM; 4 and 8 h). Mitochondrial ROS (mtROS) was detected after 4 h with 5 µM MitoSOX Red (Invitrogen) using flow cytometry.

### Intracellular farnesol staining

Monocytes were isolated and rested for 2 h on poly-D-lysin-coated µ-slides (Ibidi) and then stained with 50 µM farnesyl alcohol azide (Cayman Chemical) for 1 h at 37°C and 5% CO_2_ and clicked with 5 µM BDP-FL-PEG_4_-DBCO (Jena Bioscience) for 10 min. Mitochondria and nucleus were visualized using MitoTracker Red CMXRos (100 nM; Invitrogen) and SYTO Deep Red Nucleic Acid Stain (1 µM; Invitrogen), respectively. Cells were imaged using a spinning disk confocal microscope Ni2-E (Nikon). Images underwent background subtraction in Fiji Image J.

### Mitochondrial respiration

Untreated DCs or those differentiated in the presence of FOH (50 and 100 µM), with/without pre-treatment of PPAR-γ antagonist GW9662 (10 µM) or solvent as control, were collected after 24, 72, and 144 h, and the Mito Stress Test Assay was performed on an Extracellular Flux Analyzer, Seahorse XFe96 Pro Analyzer (Agilent Technologies).

### DC:T cell co-culture assays

DCs were differentiated in the presence of FOH (50 or 100 µM), 4-HPR (5 µM), RV (50 µM), solvent as a control, or left untreated. Alternatively, DCs were differentiated in the top chamber of a transwell insert with a *C. albicans* biofilm (SC5314 or ATCC10231) in the well below. On day 6, immature DCs were activated with 10 µg/mL Poly(I:C) HMW (InvivoGen) and Thimerosal-killed *C. albicans* SC5314 (MOI = 1) for 24 h. Allogeneic T cells were isolated from peripheral blood mononuclear cells using the Pan-T cell Isolation Kit (Miltenyi Biotec). T cells were cultured at a 1:1 ratio with mature DCs in RPMI 1640 medium containing 10% heat-inactivated human serum from male AB plasma (Sigma-Aldrich), 2 mM l-glutamine, 100 U/mL PenStrep, and 100 U/mL IL-2 (Miltenyi Biotec). After 4 days, T cells were stimulated with phorbal myristate acetate (PMA) (50 ng/mL; Sigma-Aldrich), ionomycin (1 µg/mL; Sigma-Aldrich), and brefeldin A (5 µg/mL; BioLegend) for 4 h before staining with anti-human CD3 PerCP-Vio 700 antibody (clone REA613; Miltenyi Biotec). Afterward, the cells were fixed, permeabilized, and stained intracellularly for IFN-γ (anti-human IFN-γ APC, clone 4S.B3, BioLegend) and then analyzed by flow cytometry.

### Statistical analysis

Sphingolipid and neutral lipid data were analyzed using two-way ANOVA with multiple comparison tests (Tukey’s). Other data sets were analyzed by one-way ANOVA with multiple comparison tests (Tukey’s or Dunnett’s). All statistical analyses were performed in GraphPad Prism 10.

## RESULTS

### Farnesol affects the *de novo* sphingolipid synthesis pathway

**Fig 1:**
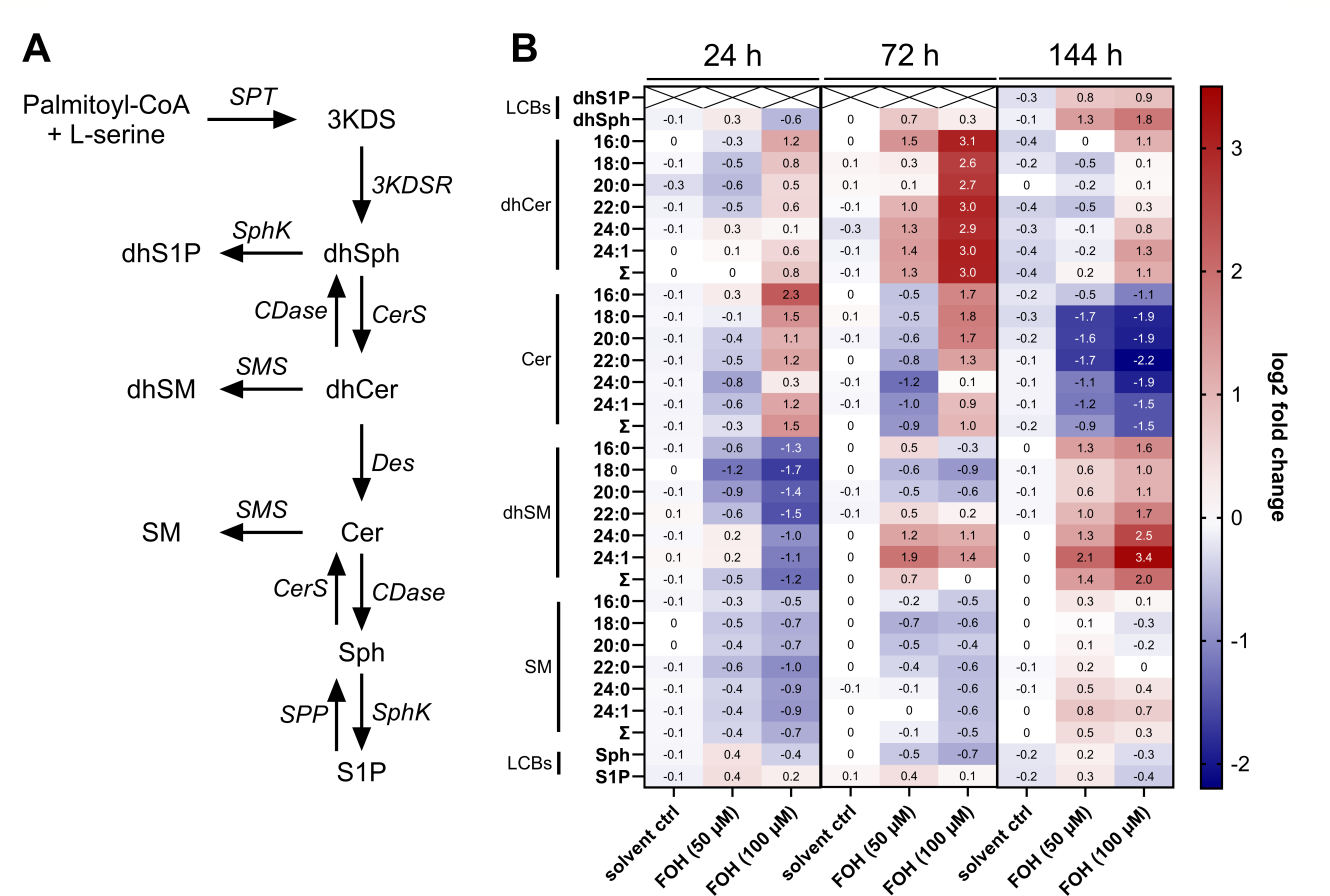
FOH affects the sphingolipid metabolism in DCs differentiated from monocytes. (**A**) The *de novo* sphingolipid synthesis pathway starts with the condensation of palmitoyl-CoA and l-serine and generates ceramide through four enzymatic steps. Cer and its precursor dihydroceramide can be converted to sphingomyelin and dihydrosphingomyelin, respectively. (**B**) Changes in sphingolipid metabolism after FOH treatment, compared to the untreated control. Monocytes were differentiated to DCs for 24, 72, and 144 h in the presence of FOH (50 and 100 µM) or solvent control. Data from at least four individual experiments (24 h, *n* = 4; 72 h, *n* = 4; and 144 h, *n* = 6) were normalized to the untreated control group, for which the log2 fold change was set to 0 (not shown in the heatmap). Statistical comparisons were performed using two-way ANOVA with Tukey’s multiple comparisons test. *X*, not detectable; dhS1P, dihydrosphingosine 1-phosphate; dhSph, dihydrosphingosine3KDSR, 3-keto-dihydrosphingosine reductase; SphK, sphingosine kinase; CerS, ceramide synthase; CDase, ceramidase; SMS, sphingomyelin synthase; and LCBs, long chain bases. *n,* the number of biological replicates.

Sphingolipids play important roles in infection and immunity, and our previous studies suggested some similarities in the signaling pathways of FOH and S1P (13, 14). We confirmed that both S1P and FOH decreased the CD1a/CD1d ratio on DCs (Fig. S1A) and that PPAR-γ is involved in FOH-associated regulation of CD1d expression (Fig. S1B). Changes to the CD1a expression and CD1a/CD1d ratio by FOH were similar when killed *C. albicans* was present (Fig. S2A) and were not induced by killed *C. albicans* alone (Fig. S2B). To determine whether FOH affects S1P levels and sphingolipid metabolism during the differentiation of DCs, we differentiated primary human monocytes to immature DCs over the course of 6 days (144 h) in the presence of FOH (50 and 100 µM) or methanol as a solvent control. Changes in sphingolipid metabolites (Fig. 1A) were analyzed at 24, 72, and 144 h using HPLC-MS/MS (Fig. 1B). However, FOH did not affect S1P levels (Fig. 1B). In contrast, the most prominent effect of FOH during DC differentiation was an increase in dihydroceramide levels. Treatment with 50 or 100 µM FOH increased dhCer levels at 72 h compared to the solvent control. Similar effects were observed at 144 h (Fig. 1B), with increased dhCer levels at 100 µM FOH. In contrast, ceramide levels were reduced at 144 h after FOH treatment. The decreased Cer/dhCer ratio (Fig. S3) pointed toward an inhibition of the last enzyme of the *de novo* synthesis pathway, dihydroceramide desaturase, which converts dhCer to Cer by adding a 4,5-*trans*-double bond (29).

Dihydrosphingomyelin species also accumulated in DCs after FOH treatment, while total sphingomyelin levels were not altered (Fig. 1B). Furthermore, FOH treatment increased dihydrosphingosine and dihydrosphingosine 1-phosphate levels at 144 h compared to the solvent control (Fig. 1B). We also measured 1-deoxysphingolipids, a rare class of sphingolipids formed by the use of l-alanine instead of l-serine by SPT (Fig. S4A). Due to the absence of a C1-OH group, 1-deoxysphingolipids cannot be used to form more complex sphingolipids such as SM (30). The levels of 1-deoxy-dhSph and 1-deoxy-dhCer were significantly increased in FOH-treated samples compared to the solvent control, while 1-deoxy-Cer levels remained unchanged (Fig. S4B). Overall, our data suggest that FOH increases SPT activity and inhibits Des activity, resulting in an accumulation of dhSph, dhS1P, dhCer, and dhSM.

### Farnesol increases serine palmitoyltransferase activity

**Fig 2:**
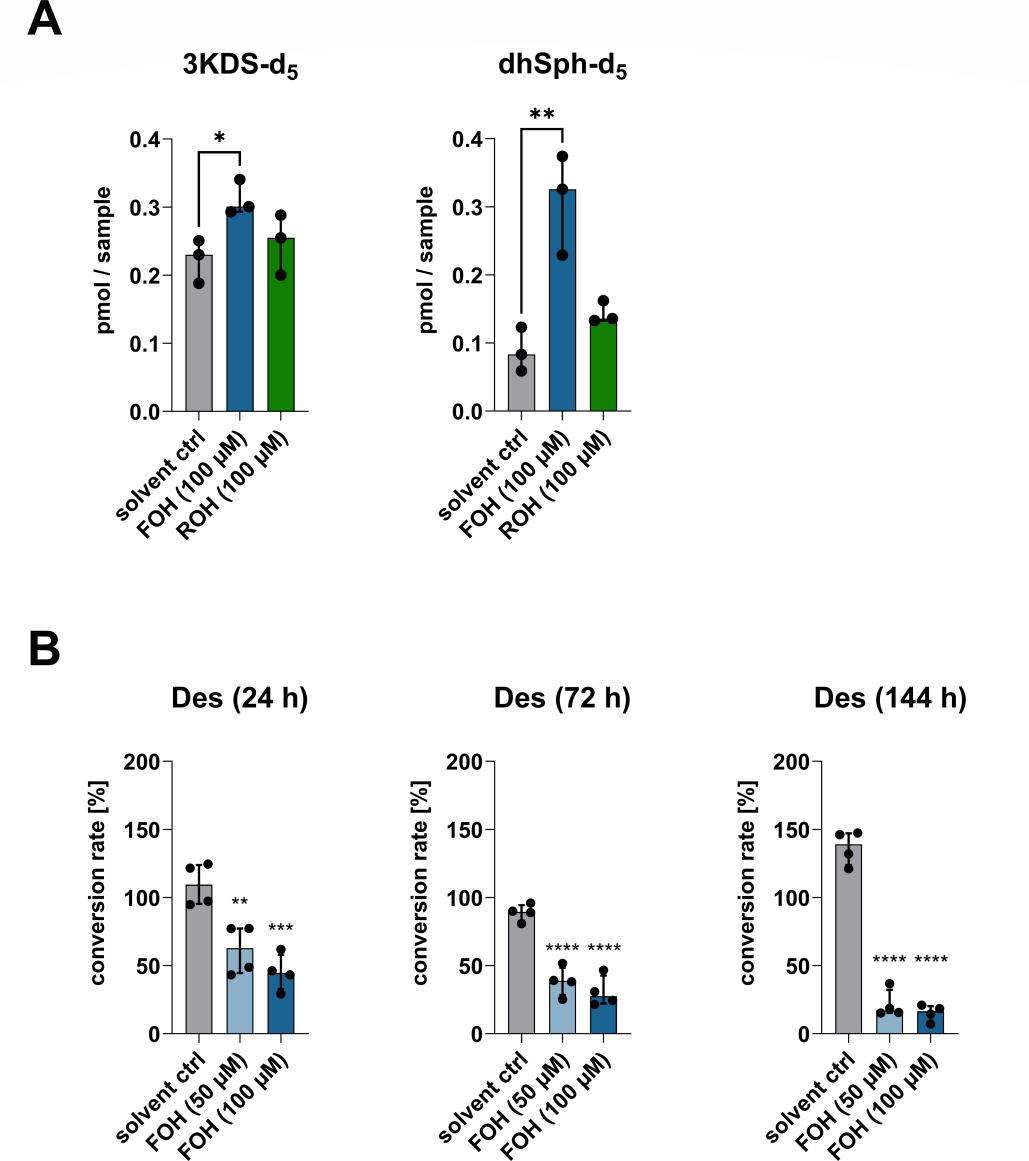
FOH modulates the sphingolipid *de novo* synthesis. (**A**) The effects of FOH (100 µM) and retinoic acid (100 µM) on 3KDS-d_5_ and dhSph-d_5_ detection in a cell-free sphingolipid *de novo* synthesis assay (applied substrates: palmitate-d_3_ and l-serine-d_3_). It should be noted that the assay for the more sensitive determination of 3KDS-d_5_ was performed without the addition of NADPH, thus stopping the *de novo* synthesis after SPT catalysis. For the quantification of the reduced downstream product, dhSph-d_5_, the assay was performed in the presence of NADPH. Data from three independent experiments were used to calculate comparisons between FOH-treated samples or ROH-treated samples and the control group. Statistical analyses were performed using one-way ANOVA with Dunnett’s multiple comparisons test. (**B**) FOH inhibits Des activity. Differentiation of monocytes to DCs was induced by GM-CSF and IL-4 in the presence of FOH (50 and 100 µM) or solvent control. Des activity was assessed after 24, 72, and 144 h by quantifying d_7_-C13:0 dhCer (substrate) and d_7_-C13:0 Cer (product) content using HPLC-MS/MS and calculating the conversion rate. Data from four independent experiments were normalized to the untreated control group, which was set to 100% (not shown in the bar chart). FOH-treated groups were compared to their solvent control using one-way ANOVA (with Dunnett’s *post hoc* test). Bars show the median with the interquartile range. *P < 0.05; **P < 0.01; ***P < 0.001; and ****P < 0.0001.

The increased levels of dhSph and dhS1P in FOH-treated cells suggested that FOH may activate SPT or 3-ketodihydrosphingosine reductase. The first product of the *de novo* sphingolipid synthesis pathway, 3KDS, is rapidly oxidized to dhSph and thus was not detectable in our cell-based assays. Therefore, we used a recently developed cell-free microsomal *de novo* sphingolipid synthesis assay (20) to detect 3KDS-d_5_ as a direct measure of SPT activity (condensation of d_3_-palmitoyl-CoA and l-serine-d_3_; loss of one deuterium label during catalysis). 3KDS-d_5_ and dhSph-d_5_ levels were significantly increased in the presence of FOH (100 µM) compared to the solvent control, indicating that FOH increases SPT activity (Fig. 2A). As retinoic acid (ROH) is also known to activate SPT (31), as well as PPAR-γ, we additionally tested the effect of ROH (100 µM) on SPT, but in the human microsome assay, it did not significantly increase 3KDS-d_5_ or dhSph-d_5_ levels (Fig. 2A). Furthermore, we used a dose-response assay for FOH and ROH and found distinct effects for both substances on the sphingolipid *de novo* synthesis (Fig. S5).

### Farnesol inhibits dihydroceramide desaturase activity

The analysis of the cellular sphingolipidome in DCs differentiated from monocytes showed that FOH treatment led to an accumulation of dhCer and dhSM species and a reduction in Cer, suggesting the inhibition of Des. Therefore, we aimed to directly quantify the effects of FOH on Des in DCs by measuring the intracellular conversion of d_7_-C13:0 dhCer (Des substrate) to d_7_-C13:0 Cer (Des product). There was no reduction in Des activity 1, 6, or 12 h after incubation with FOH (Fig. S6A). After 24 h, mean Des activity was reduced to ~60% in monocytes differentiating into DCs treated with 50 µM FOH and to ~45% in cells treated with 100 µM FOH, compared to the solvent control (Fig. 2B). Enzyme activity continued to decrease at later time points, with an activity of ~15% at 100 µM FOH after 144 h, when monocytes had differentiated into DCs (Fig. 2B). *DEGS1* gene expression was not affected by FOH treatment, suggesting that regulation was not at the transcriptional level (Fig. S6B). Furthermore, we could exclude the involvement of FOH-dependent PPAR-γ activation in the regulation of Des (Fig. S6C). Thus, FOH treatment inhibits Des activity during DC differentiation, explaining the decreased Cer/dhCer ratio and accumulation of dhSM.

### Farnesol induces ROS through an NADPH-independent mechanism

**Fig 3:**
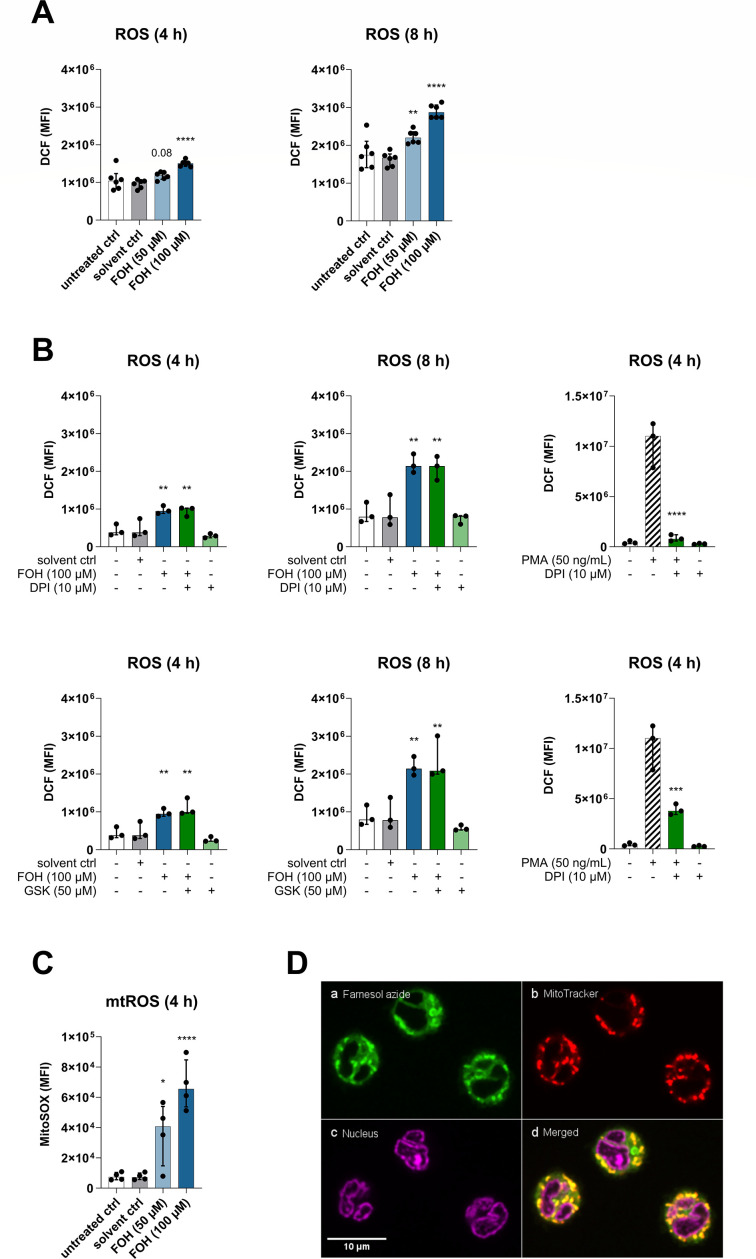
FOH induces ROS independent of NADPH oxidases. Monocytes were treated with GM-CSF and IL-4 to induce differentiation into DCs in the presence of FOH (50 or 100 µM), solvent control, or left untreated. (**A**) ROS levels were determined using DCF (10 µM) after 4 and 8 h in FOH-treated cells or control groups (*n* = 6). (**B**) Cells were treated with NOX inhibitors DPI (10 µM) or GSK (50 µM) for 1 h prior to the addition of FOH or for 4 h on control cells. PMA (50 ng/mL) was used as a positive control to test the efficacy of DPI and GSK. ROS was determined using the DCF assay (*n* = 3). (**C**) Mitochondrial ROS was detected at 4 h by flow cytometry using the MitoSOX stain (*n* = 4). (**D**) (a) Farnesyl alcohol azide (50 µM) was clicked with BDP-FL-PEG_4_-DBCO (5 µM) and visualized in freshly isolated monocytes using a spinning disk confocal microscope. (b) Mitochondria were visualized using MitoTracker (100 nM) and (c) nuclei were stained with SYTO Deep Red Nucleic Acid Stain (1 µM). (d) The merged image shows the co-localization of clickable azido-FOH and mitochondria. Statistical comparisons between groups were performed using one-way ANOVA with Dunnett’s multiple comparison test (ns, not significant; *P < 0.05; **P < 0.01; ***P < 0.001; and ****P < 0.0001), and differences compared to the solvent control are displayed. *n,* the number of individual donors (biological replicates). Bars show the median with an interquartile range.

Oxidative stress inhibits Des activity *in vitro* and *in vivo*, leading to the accumulation of dhCer (32). Because FOH induces the production of ROS in various fungal species and immune cells (13, 33–37), we hypothesized that the inhibition of Des is mediated indirectly by FOH-induced ROS. Thus, we measured intracellular levels of ROS in FOH-treated cells. Monocytes differentiating into DCs treated with 100 µM FOH showed increased levels of ROS as early as 4 h post-treatment, while cells treated with 50 µM FOH had higher ROS levels after 8 h (Fig. 3A). Next, we attempted to inhibit FOH-induced ROS production to determine whether the inhibition of Des was linked to oxidative stress. We blocked NADPH oxidases (NOX), which are key enzymes in the generation of ROS during inflammation and immunity (38), using the NOX inhibitor DPI and the NOX2 inhibitor GSK2795039. The inhibitors were added 1 h prior to treatment with FOH, and induction of ROS by PMA was used as a positive control to test the activity of the inhibitors. While PMA-induced ROS could be inhibited by DPI and GSK, neither inhibitor affected the levels of FOH-induced ROS (Fig. 3B). This suggests that FOH induces ROS production by a NOX-independent mechanism in monocytes differentiating to DCs.

### Farnesol induces mitochondrial ROS

Mitochondria are a major source of cellular ROS (39). We tested whether FOH could induce the production of mtROS by flow cytometry, using MitoSOX. Both 50 and 100 µM FOH increased mtROS in monocytes differentiating into DCs as early as 4 h post-treatment, with 100 µM FOH causing an almost ninefold increase compared to the solvent control (Fig. 3C). Thus, the mitochondrial respiratory chain seems to be an important source of FOH-induced ROS.

It has not been shown whether FOH can enter the host immune cell or signals via binding to surface receptors, but due to the strong effect of FOH on mtROS, we suspected that FOH may enter the cell. Thus, we investigated the intracellular localization of FOH using a modified version of FOH containing an azido group, which could be fluorescently labeled with BDP-FL-PEG_4_-DBCO by click chemistry. Azido-FOH was broadly distributed inside the cell, but there seemed to be an accumulation at the mitochondria (Fig. 3D). Co-staining with MitoTracker demonstrated clear co-localization of clicked azido-FOH at the mitochondria, showing that there is a potential for a direct interaction between FOH and mitochondria.

### Farnesol-induced ROS generation inhibits dihydroceramide desaturase activity

**Fig 4:**
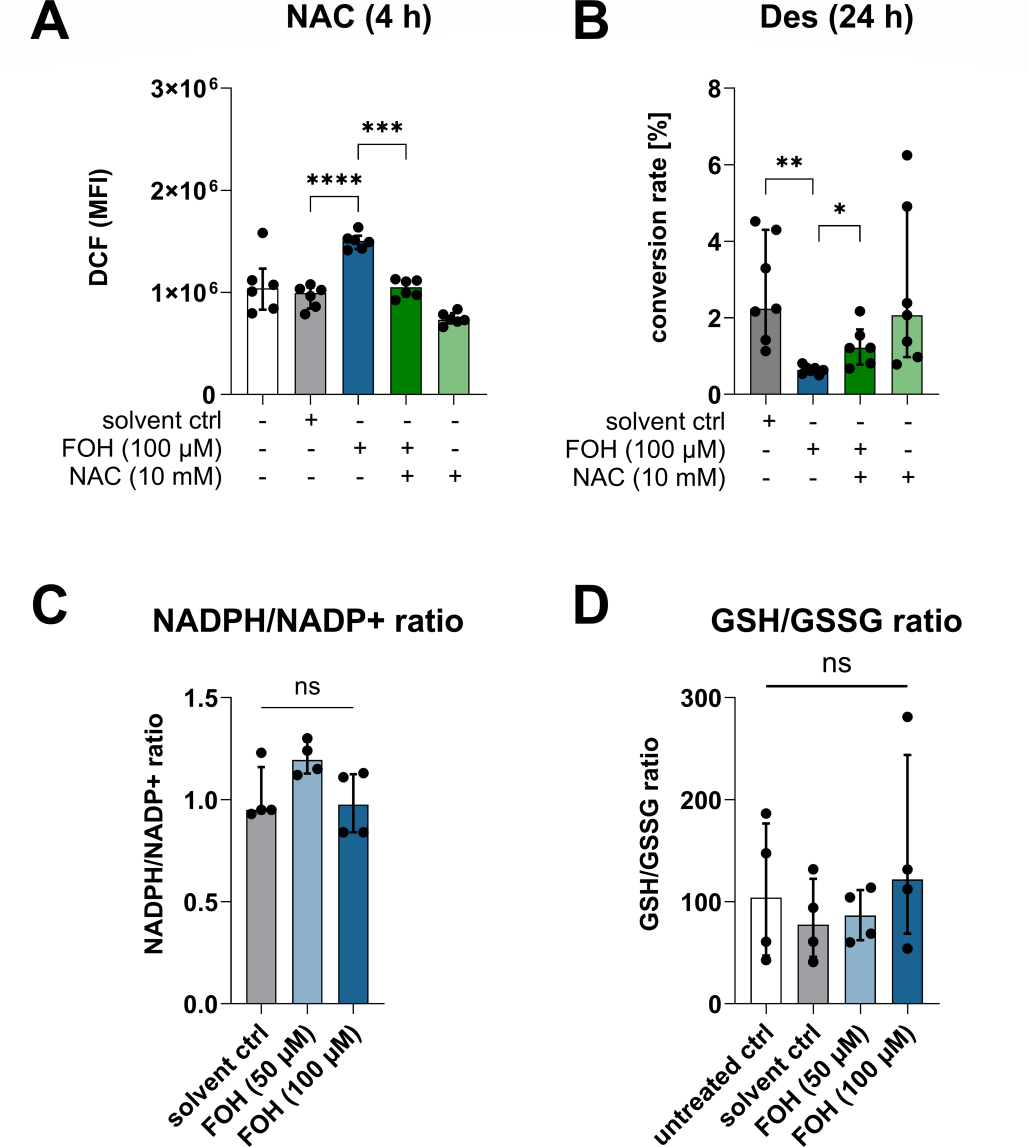
FOH-induced ROS production inhibits Des activity. (**A**) Generation of ROS in differentiating monocytes was determined after 4 h using the DCF assay (*n* = 6). The antioxidant NAC (10 mM) was able to neutralize ROS induced by 100 µM FOH over 4 h. (**B**) Des activity was measured by HPLC-MS/MS after 24 h in untreated cells and cells treated with FOH (100 µM) and NAC (10 mM) alone or in combination (*n* = 7). Comparison to FOH-treated cells was performed using one-way ANOVA with Tukey’s post-comparison test. (**C**) NADPH/NADP+ ratio in FOH- and solvent control-treated DCs (144 h; *n* = 4). Data were normalized to the untreated control group, which was set to 1 (not shown in the bar chart). (**D**) GSH/GSSG ratio in DCs (144 h) treated with FOH (50 and 100 µM), solvent control, or left untreated (*n* = 4). Bars show the median with an interquartile range; not all statistical comparisons are displayed (ns, not significant; *P < 0.05; **P < 0.01; ***P < 0.001; and ****P < 0.0001). *n,* the number of biological replicates.

Next, we aimed to determine whether inhibiting FOH-induced mtROS could restore Des activity. In *Saccharomyces cerevisiae*, myxothiazol, which inhibits the Rieske iron-sulfur center of the mitochondrial complex III, was most effective at reducing FOH-induced ROS (35). However, neither myxothiazol nor any of the other individual mitochondrial complex inhibitors we tested (rotenone: complex I; antimycin A and S3QEL 2: complex III; and oligomycin: complex V) could prevent FOH-induced generation of ROS in monocytes differentiating to DCs (Fig. S7A through E). The involvement of PPAR-γ in FOH-dependent regulation of ROS could also be excluded (Fig. S7F). We attempted to neutralize FOH-induced ROS by treating cells with the antioxidant *N*-acetyl-l-cysteine. When given together with FOH, NAC reduced ROS levels at 4 h compared to FOH alone (Fig. 4A). Thus, we used NAC to determine whether FOH-induced ROS was responsible for the inhibition of Des (Fig. 4B). After 24 h, FOH-mediated inhibition of Des could be prevented by treatment with NAC, demonstrating a link between oxidative stress and the inhibition of Des by FOH. However, FOH did not alter NADPH or glutathione pools in differentiated DCs (Fig. 4C and D).

### Farnesol impairs mitochondrial respiration and alters triacylglycerol synthesis in monocytes differentiating to DCs

**Fig 5:**
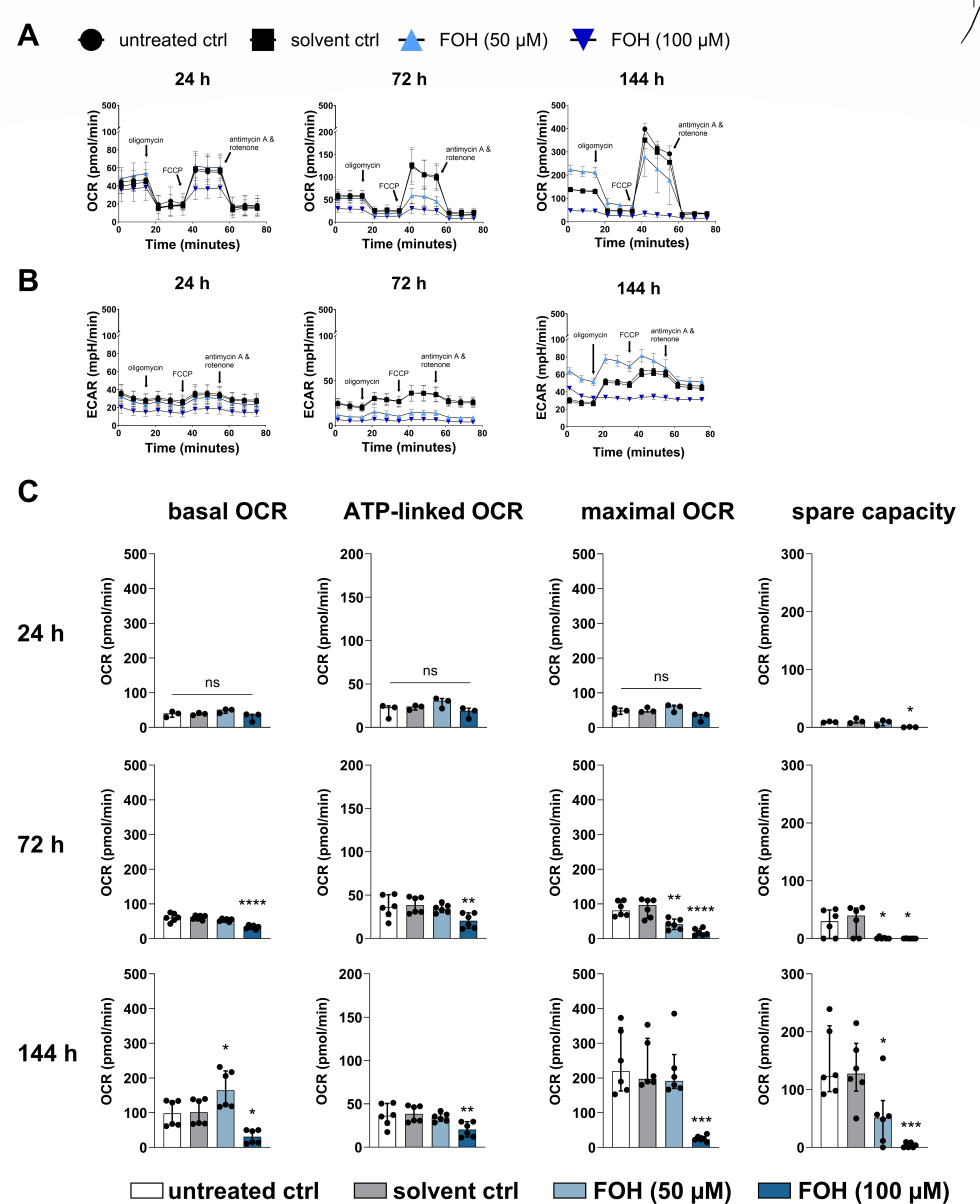
FOH impairs mitochondrial respiration during differentiation of monocytes to DCs. Monocytes were given GM-CSF and IL-4 to induce DC differentiation and treated with FOH (50 and 100 µM) or solvent control or left untreated for 24 h (*n* = 3), 72 h (*n* = 6), and 144 h (*n* = 6). The Seahorse XF Cell Mito Stress Test was performed to evaluate mitochondrial respiration by measuring the oxygen consumption rate and the extracellular acidification rate. (**A**) Representative OCR curve for each time point. (**B**) Representative ECAR curve for each time point. (**C**) Differences between treatment groups were calculated by one-way ANOVA with Tukey’s *post hoc* test for the basal respiratory OCR, the maximal respiratory OCR induced by the uncoupler FCCP (1 µM), the spare respiratory capacity, determined by the difference between maximal and basal OCR and ATP-linked OCR, determined following the inhibition of ATP synthesis using oligomycin (2 µM). ns, not significant; *P < 0.05; **P < 0.01; ***P < 0.001; and ****P < 0.0001.

A recent study found that deficiency in Des due to mutations in the *DEGS1* gene led to impaired mitochondrial respiration (40). Therefore, we investigated the effects of FOH on mitochondrial respiration to explore whether the inhibition of Des and subsequent increase in dhCer during the course of monocyte differentiation into DCs coincided with an effect on mitochondrial function. We analyzed oxygen consumption rate (OCR) as a measure of mitochondrial respiration and extracellular acidification rate (ECAR) as a measure of glycolysis by performing the Seahorse Mito Stress Test at 24, 72, and 144 h into the differentiation process (Fig. 5A and B). Basal OCR was reduced in FOH-treated cells at 72 and 144 h, but not at 24 h, compared to the solvent controls, indicating that overall mitochondrial respiration was reduced over time (Fig. 5C). Furthermore, the maximal OCR after the addition of Carbonyl cyanide-p-trifluoromethoxyphenylhydrazone (FCCP) was lower in FOH-treated cells compared to controls (Fig. 5C). The difference between basal and maximal respiration constitutes the spare capacity, also known as the mitochondrial reserve, and indicates the capacity of the cell to respond to increased energy demands (41). The spare capacity was reduced in FOH-treated cells compared to solvent-treated cells as early as 24 h into the differentiation process at 100 µM FOH and by 72 h at 50 µM FOH (Fig. 5C). While the ECAR was decreased at 24 and 72 h, ECAR measurements were similar between FOH-treated cells and controls in fully differentiated immature DCs (144 h) (Fig. S8A). Thus, the OCR/ECAR ratio was reduced at 144 h (Fig. S8B). In summary, FOH reduces the mitochondrial reserves in monocytes differentiating into DCs, which would hinder their ability to adapt metabolically to an increased energy demand. The ATP-linked OCR was decreased in FOH (100 µM)-treated cells after 72 h, suggesting a reduced production of mitochondrial ATP (Fig. 5C); however, overall cellular ATP levels remained unchanged in the presence of FOH (Fig. S9A). Furthermore, the mitochondrial membrane potential (Δψm; Fig. S9B) and mitochondrial mass (Fig. S9C) remained unchanged in the presence of FOH. The effect of FOH on mitochondrial function did not affect DC viability (Fig. S9D), induced autophagy (as measured by LC3 staining) (Fig. S9E), or changed phagocytosis of *C. albicans* by DCs (Fig. S9F).

**Fig 6:**
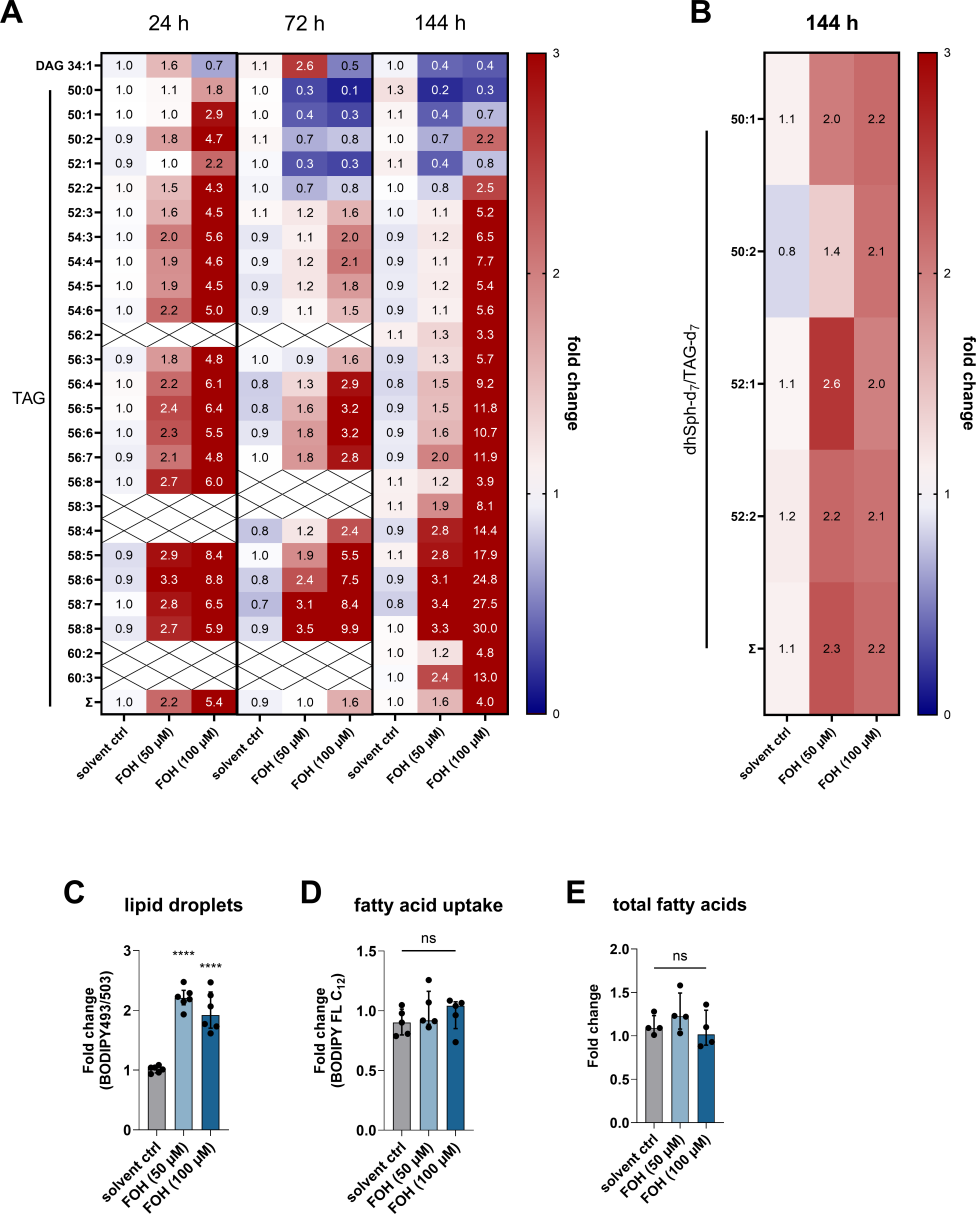
FOH induces triacylglycerol accumulation and lipid droplet formation in DCs. (**A**) Diacylglycerol (34:1) and triacylglycerol synthesis during differentiation of monocytes to DCs in the presence of FOH (50 and 100 µM) or solvent control was measured by UPLC-qTOF-MS. Fold change was calculated relative to the untreated control group (set to 1; not shown) for four independent experiments (*n* = 4). (**B**) DCs were differentiated for 144 h in the presence of FOH (50 and 100 µM) or solvent control and fed with d_7_-dhSph (1 µM for 4 h). Individual d_7_-TAG species were quantified, and the d_7_-dhSph/d_7_-TAG ratio was calculated. Fold change was calculated compared to the untreated control group (set to 1; not shown) for four independent experiments (*n* = 4). (**C**) Lipid droplet formation was detected by BODIPY493/503 in FOH-treated DCs (144 h; *n* = 6) by flow cytometry. (**D**) Fatty acid uptake was quantified by BODIPY FL C_12_ staining in DCs (144 h; *n* = 5) using flow cytometry. Fold change was calculated relative to the untreated control group (set to 1; not shown in the bar diagram). (**E**) Total cellular fatty acids quantified in FOH- and solvent control-treated DCs (144 h) using UPLC-qTOF-MS. Fold change was calculated relative to the untreated control group (set to 1; not shown in the bar diagram). ns, not significant and ****P < 0.0001.

Deficiencies in mitochondrial function and the disruption of mitochondrial-associated membranes by the loss of Des function in patients with a mutation of *DEGS1* are associated with an increase in lipid droplet formation, corresponding with an increase in the activity of DGAT2, an enzyme that converts diacylglycerols (DAG) to triacylglycerols (TAG) (40). During DC differentiation, FOH increased cellular TAG content (Fig. 6A) and increased lipid droplet formation (Fig. 6C), while reducing cellular DAG levels (Fig. 6A). Using deuterium-labeled d_7_-dhSph, we could show that increased TAG levels were associated with FOH-induced alterations in sphingolipid metabolism (Fig. 6B). As the only irreversible step out of the complex sphingolipid metabolism network, S1P lyase cleaves dhS1P or S1P into hexadecenal and phosphoethanolamine. The fatty aldehyde dehydrogenase (also known as ALDH3A2) converts the released fatty aldehydes into the corresponding fatty acids, which, after activation by acyl-CoA synthetases, can be used as building blocks for the synthesis of glycerolipids (such as DAG and TAG) (42). For our study, this indicates that d_7_-dhSph, which was added to the cells, is only insufficiently incorporated into ceramides due to Des inhibition. Instead, more d_7_-dhS1P is formed. After the mechanism described above, the S1P lyase releases d_7_-hexadecanal, which can be found as a d_7_-palmitate side chain in TAGs after oxidation and activation. In support of this, fatty acid uptake and total fatty acid content remained unchanged in the DCs across different treatment conditions (Fig. 6D and E). Furthermore, PPAR-γ activation did not influence FOH-induced alterations in mitochondrial respiration and TAG synthesis (Fig. S10).

### Inhibition of dihydroceramide desaturase impairs interferon secretion by DCs and T cells

**Fig 7:**
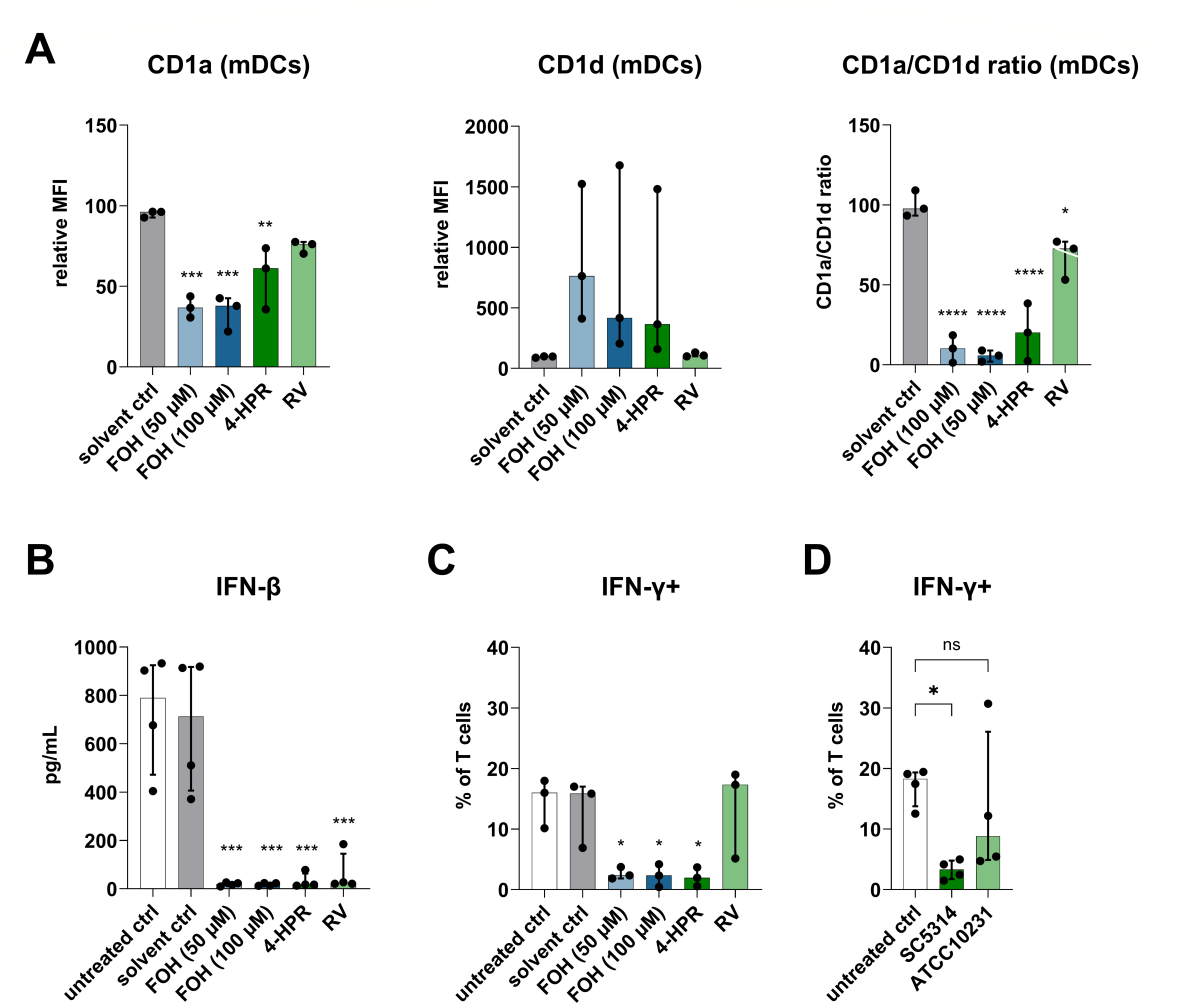
Immunomodulatory effects of Des inhibition. (**A**) CD1a and CD1d expression of mature DCs. DCs were differentiated in the presence of methanol as a solvent control, FOH (50 and 100 µM), 4-HPR (5 µM), or RV (50 µM) and matured for 24 h with LPS (100 ng/mL). Data from three individual experiments were normalized to the untreated control (set to 100%; not shown in the bar diagrams). (**B**) IFN-β secretion by DCs differentiated in the presence of solvent (control), FOH (50 and 100 µM), 4-HPR (5 µM), or RV (50 µM) after maturation with LPS (100 ng/mL) for 24 h (*n* = 4). Intracellular IFN-γ staining in T cells activated by (**C**) mature DCs differentiated in the presence of solvent (control), FOH (50 and 100 µM), 4-HPR (5 µM), RV (50 µM), or left untreated (*n* = 3) and (**D**) mature DCs differentiated in transwell inserts on top of *C. albicans* SC5314 and ATCC10231 biofilms (*n* = 4). DCs were matured in the presence of 10 µg/mL Poly(I:C) HMW and Thimerosal-killed *C. albicans* SC5314 (MOI = 1) for 24 h. ns, not significant; *P < 0.05; **P < 0.01; ***P < 0.001; and ****P < 0.0001.

Previously, we have shown that FOH alters surface marker expression and cytokine secretion in DCs, resulting in reduced T cell priming and interferon-γ secretion (13, 14). To test the relevance of Des in DC function, we used two commonly used Des inhibitors: fenretinide (5 µM) and resveratrol (50 µM). When administered at day 5 during the DC differentiation protocol for 24 h, both inhibitors, similarly to FOH, reduced the CD1a/CD1d ratio in mature DCs (Fig. 7A). Since mitochondrial dysfunction and release of mitochondrial ROS can activate the inflammasome and inflammatory immune responses, leading to the release of cytokines such as IL-1β and type I interferons, we additionally measured IL-1β and IFN-β release by DCs differentiated in the presence of FOH in response to LPS. IFN-β secretion by mature DCs was diminished after FOH (50 or 100 µM), 4-HPR, or RV treatment (Fig. 7B), whereas IL-1β secretion remained unchanged (Fig. S11). Furthermore, DCs treated with FOH (50 or 100 µM) or 4-HPR could no longer induce IFN-γ production by allogeneic primary human T cells (Fig. 7C; Fig. S12).

Finally, to test the effect of FOH in a setting closer to *in vivo* condition*s*, we established a *C. albicans* biofilm in the bottom compartment of transwell plates using either the wild-type SC5314 *C. albicans* or ATCC10231, a FOH-deficient strain, and differentiated DCs from monocytes in the transwell insert. DCs differentiated in the presence of the SC5314 strain could not induce sufficient IFN-γ production in T cells compared to control DCs cultured in the absence of *C. albicans*, while DCs differentiated in the presence of the FOH-deficient ATCC10231 strain could induce IFN-γ production in T cells similarly compared to control DCs (Fig. 7D). These results confirm that FOH produced by *C. albicans* in a biofilm alters the ability of DCs to induce IFN-γ-producing T cells similarly to exogenously added FOH and Des inhibitors.

## DISCUSSION

The opportunistic fungal pathogen *C. albicans* produces FOH as a quorum-sensing molecule. This lipophilic alcohol inhibits the yeast-to-hypha transition and the formation of biofilms, processes that are important in the virulence of *C. albicans* (43). Importantly, FOH is also involved in cross-species and even cross-kingdom communication. It plays an important role in the interactions of *C. albicans* with its host, the microbiome, and competing pathogens. Recently, we showed that FOH affects the sentinels of the immune system, DCs. Using primary human monocytes differentiating into DCs, we found that FOH altered the expression of antigen presentation and maturation markers, reduced the secretion of IL-12 while increasing the expression of IL-10, and impaired the ability of DCs to prime T cells (13, 14). Here, we expand upon those results by demonstrating that FOH has strong effects on sphingolipid metabolism.

The *de novo* synthesis of sphingolipids occurs at the outer leaflet of the ER, where a set of four enzymes generates Cer, which can be shuttled to the Golgi apparatus to produce more complex sphingolipid species (44). The first enzyme of the *de novo* synthesis pathway, SPT, catalyzes the condensation of l-serine and palmitoyl-CoA to form 3KDS (45). In our study, FOH increased the levels of 3KDS and dhSph, indicating that it increased SPT activity. The accumulation of dhSph consequently led to an accumulation of dhS1P. Using a cell-free microsomal assay (20), we subsequently found that FOH seems to directly activate SPT. Interestingly, it has also been reported that ROH activates SPT (31), although we were unable to confirm these effects in the human microsome assay. ROH is also a ligand for RARα and PPAR-γ, nuclear receptors, which mediated the FOH-induced increase in CD1d on DCs. FOH metabolites are required for the synthesis of retinoids via the mevalonate pathway. Furthermore, FOH and related metabolites were identified as activating ligands of a heterodimeric complex formed by retinoid X receptor (RXR) and farnesoid X-activated receptor in mammals, and both RARα and PPARs form heterodimers with RXR (46). FOH has been shown to activate both PPAR-γ and PPAR-α (47, 48). Thus, there appear to be multiple links between FOH, retinoid receptors, and PPAR-γ.

Our second important finding was that FOH inhibits Des activity in monocytes differentiating to DCs. Des is the last enzyme in the *de novo* sphingolipid synthesis pathway and converts dhCer to Cer by inserting a 4,5-*trans*-double bond (29). Thus, the inhibition of Des by FOH led to the accumulation of dhCer species and reduced levels of Cer. An increase in the dhCer/Cer ratio increases membrane rigidity (49). It has been reported that accumulation of Cer induces apoptosis (50, 51), while accumulation of dhCer leads to autophagy (52). However, we did not see an effect on viability, apoptosis, or autophagy in our cells when dhCer was increased due to FOH treatment. It has also been reported that an increase in dhCer levels causes a metabolic switch from SM to dhSM production (53). This was reflected in our data, as we found an accumulation of dhSM species after FOH treatment, while the SM levels remained unchanged. As dhSM plays a role in membrane-related processes, Des plays an important role in some infections by modulating dhSM levels (53, 54). For example, the efficacy of two polyphenol inhibitors against flaviviruses, including West Nile virus, dengue, Usutu, and Zika viruses, was due to their inhibition of Des, leading to increased dhSM (53). In phospholipid:sphingolipid:cholesterol mixtures, SM promotes fluid domains, while dhSM tends to form rigid domains (54). As rigid membranes are more resistant to the insertion of the HIV gp41 fusion peptide, it is possible to inhibit HIV-cell membrane fusion by inhibiting Des, leading to an increase in dhSM (54). Although SM is known to be an important molecule in the phagocytosis or invasion of many bacteria (22, 55), to the best of our knowledge, the role of Des and dhSM in these processes has not been examined in either bacterial or fungal infections.

Des inhibition by FOH in DCs was induced by the generation of ROS. It is well-established that ROS and oxidative stress inhibit Des activity in a time- and dose-dependent manner, without changing the protein levels of Des (32). Pharmaceutical inhibitors, such as fenretinide (4-HPR), a synthetic retinoid derivative, inhibit Des activity by inducing ROS (47). It has been suggested that inhibition is related to changes in cellular redox status and NADPH levels or the depletion of cellular thiol (32). Des requires NADH/NADPH as electron donors and oxygen as an electron acceptor for its activity, generating NAD^+^/NADP^+^ (29). We found that FOH induced ROS in monocytes differentiating to DCs but FOH did not change the NADPH or glutathione pool. Previously, we observed that FOH induced the oxidative burst in neutrophils (13), and increased ROS production has also been observed in murine macrophages treated with FOH (33). Interestingly, FOH also induces ROS in *C. albicans* itself (37), but simultaneously protects *C. albicans* against oxidative stress (56). However, FOH inhibits the growth of another yeast, *S. cerevisiae*, by inducing ROS (34–37). In *S. cerevisiae*, FOH induced mtROS, correlating with hyperpolarization of the mitochondrial transmembrane potential (34). Interestingly, in *S. cerevisiae*, FOH could accelerate ROS generation even under conditions in which mitochondrial electron transport was fully repressed, possibly by utilizing the mitochondrial electron flow from the remaining ubiquinol pool. This mtROS was most effectively inhibited by myxothiazol, which inhibits the Rieske iron-sulfur center of complex III (35). Leakage of electrons from complex III of the mitochondrial respiratory chain is estimated to be responsible for ~80% of ROS normally produced in cells (57). We found a similar increase in mtROS after FOH treatment of monocytes differentiating into DCs. However, we were unable to inhibit FOH-induced ROS production using myxothiazol or any of the other individual mitochondrial complex inhibitors we tested. However, the use of the antioxidant NAC successfully prevented FOH-induced ROS production.

Mitochondrial respiration declined over time in DCs differentiated in the presence of FOH. While mitochondria are one of the main sources of cellular ROS, high levels of ROS can in turn affect mitochondrial function (39). In addition to being present in the ER, Des has also been detected at the mitochondria (58), where it was found to affect lipid metabolism in a recent study (40). *DEGS1*-deficient fibroblasts showed altered mitochondrial dynamics and morphology, as well as higher levels of mtROS and cellular ROS than control cells. In addition, *DEGS1* deficiency was associated with changes in the synthesis of neutral lipids, resulting in increased lipid droplet formation (40). Based on this study, which showed that increased dhCer caused by Des deficiency led to a decline in mitochondrial function (40), we speculate that the reduced mitochondrial respiration we observed after FOH treatment may be due to the accumulation of dhCer. Additionally, we also found an accumulation of TAG and increased lipid droplet formation after FOH treatment. As FOH treatment also led to increased levels of 1-deoxysphingolipid species, it is worth noting that exogenous feeding of 1-deoxy-dhSph to different cell lines causes deficiencies in mitochondrial respiration, a reduction in ATP synthesis and changes in mitochondrial morphology (59). However, it is also possible that the effects of FOH on mitochondrial respiration in our cells were independent of dhCer. In *S. cerevisiae*, the addition of FOH inhibited mitochondrial electron transport and restricted cellular oxygen consumption almost immediately, despite the fact that these effects appeared not to be due to direct inhibition by FOH, but rather dependent on protein kinase C (PKC) (35). More recent studies have confirmed a role for the PKC1 signaling pathway in the regulation of FOH-induced mtROS (57). Therefore, it is also possible that the effects of FOH on mitochondrial respiration in our cells were independent of dhCer.

Finally, we show novel data demonstrating that inhibition of Des has functional consequences in DCs. Previously, we found that FOH decreased the CD1a/CD1d ratio in DCs and that this was linked to PPAR-γ activation (13, 14). In the present study, we confirmed these results and additionally show that inhibition of Des is linked to the decreased CD1a/CD1d ratio. This adds to previously published data that describe altered expression patterns of costimulatory molecules on DCs by the Des inhibitor RV (60, 61). In addition, both FOH and the Des inhibitors 4-HPR and RV diminished the secretion of IFN-β by mature DCs. IFN-β contributes to the activation of DCs and regulates IFN-γ production by Th1 cells (62). Furthermore, inhibition of Des by either FOH or 4-HPR led to a striking decrease in the ability of DCs to induce IFN-γ-producing T cells. Interestingly, RV did not have the same effects as 4-HPR. 4-HPR, a retinol derivative that shows structural similarities to FOH, inhibits Des by the generation of ROS, similar to FOH (63). In contrast, the antioxidant RV is thought to inhibit Des activity indirectly by altering the redox state of the cells (63). The inhibitory effect of FOH on the ability of DCs to induce IFN-γ-producing cells could be recapitulated in a transwell system containing a biofilm of wild-type *C. albicans* SC5314 or a FOH-deficient *C. albicans* strain, ATCC10231, that produces farnesoic acid instead of FOH (64). Here, DCs differentiated in the presence of the SC5314 strain reduced IFN-γ production by T cells, whereas DCs differentiated in the presence of the FOH-deficient ATCC10231 strain did not reduce IFN-γ production by T cells. In a mouse model of disseminated *C. albicans* infection, FOH-deficient strains had attenuated virulence (65), suggesting that FOH levels secreted by *C. albicans in vivo* are also sufficient to cause phenotypic differences.

In summary, our results show that FOH modulates sphingolipid metabolism by enhancement of SPT activity, resulting in elevated levels of 3KDS, dhSph, and dhS1P and by inhibition of Des activity via induction of ROS, leading to the accumulation of dhCer and dhSM. Furthermore, FOH treatment caused a gradual decline in mitochondrial respiration and an increase in TAG levels and lipid droplet formation. In addition, we showed that inhibition of Des affects the phenotype and function of DCs. While previously we showed the involvement of PPAR-γ in the increased expression of CD1d by FOH (14), here we show a new molecular mechanism of FOH in regulating sphingolipid and mitochondrial metabolism, independent of PPAR-γ. This cross-kingdom communication reveals a new way in which *C. albicans* may manipulate host cells, reducing protective IFN responses.

## SUPPLEMENTAL MATERIAL

Supplemental Material mbio.00732-24-s0001.pdf Supplemental methods, table, and figures.

ASM does not own the copyrights to Supplemental Material that may be linked to, or accessed through, an article. The authors have granted ASM a non-exclusive, world-wide license to publish the Supplemental Material files. Please contact the corresponding author directly for reuse.
