# Supplementary material for: The Candida albicans quorum-sensing molecule farnesol alters sphingolipid metabolism in human monocyte-derived dendritic cells
Source: mBio. 2024 Jul 2;15(8):e00732-24. doi: 10.1128/mbio.00732-24 (PMC11323541; doi:10.1128/mbio.00732-24)
Supplement: Supplemental Material — Supplemental methods, table, and figures. [file mbio.00732-24-s0001.pdf]

SUPPLEMENTAL MATERIAL

The *Candida albicans* quorum-sensing molecule farnesol alters sphingolipid metabolism in human monocyte-derived dendritic cells

Maria Batliner,[1] Fabian Schumacher,[2] Dominik Wigger,[2] Wolfgang Vivas,[3,4,5] Agata Prell,[2] Ingo Fohmann,[1] Tobias Köhler,[1] Rebekka Schempp,[6] Angela Riedel,[7] Martin Vaeth,[8] Agnes Fekete[9], Burkhard Kleuser,[2] Oliver Kurzai,[1,10,11,*] and Natalie E. Nieuwenhuizen[1]

[1]Institute for Hygiene and Microbiology, Julius-Maximilians University of Würzburg, Würzburg, Germany

[2]Institute of Pharmacy, Freie Universität Berlin, Berlin, Germany

[3]Institute for Infectious Diseases and Infection Control, Jena University Hospital – Friedrich Schiller University, Jena, Germany

[4]Associated Research Group Translational Infection Medicine, Leibniz Institute for Natural Product Research and Infection Biology - Hans Knoell Institute (HKI), Jena, Germany

[5]Department of Anesthesiology and Intensive Care Medicine, Jena University Hospital - Friedrich Schiller University, Jena, Germany

[6]Institute for Virology and Immunobiology, Julius-Maximilians University of Würzburg, Würzburg, Germany

[7]Mildred Scheel Early Career Center (MSNZ), University Hospital of Würzburg, Würzburg, Germany

[8]Würzburg Institute of Systems Immunology, Max Planck Research Group, Julius-Maximilians University of Würzburg, Würzburg, Germany

[9]Pharmaceutical Biology, Julius-von-Sachs-Institute, Biocenter, University of Würzburg, Würzburg, Germany

[10]Research Group Fungal Septomics, Leibniz Institute for Natural Product Research and Infection Biology – Hans Knoell Institute, Jena, Germany

[11]National Reference Center for Invasive Fungal Infections, Leibniz Institute for Natural Product Research and Infection Biology – Hans Knoell Institute, Jena, Germany

*Corresponding author: oliver.kurzai@uni-wuerzburg.de

SUPPLEMENTARY METHODS

Preparation of farnesol

Trans,trans-farnesol (FOH) was obtained as 4 M stock solution from Sigma-Aldrich and diluted to a 1 M solution in 100% methanol. Working concentrations of 50 µM and 100 µM were prepared in RPMI 1640 supplemented with 5% heat inactivated FBS, as described previously (1).

Cell-free sphingolipid *de novo* synthesis assay

The effects of FOH and retinoic acid (ROH) on the sphingolipid *de novo* synthesis were investigated using a microsomal assay that we recently established (2), using human liver microsomes. Briefly, the assay mixture contained 200 µg human liver microsomes as source of ER-bound sphingolipid metabolizing enzymes (SPT, 3KDSR, CerS and Des; see Fig. 1A), palmitate-d_3 (Cortecnet) and L-serine-d_3 (CDN Isotopes) as deuterated substrates of sphingolipid *de novo* synthesis as well as all required co-factors, reducing equivalents and buffers. After addition of FOH or ROH, assay mixtures were incubated for 1 h at 37°C under gentle shaking (120 rpm). A corresponding volume of methanol served as vehicle control. Reactions were stopped by addition of methanolic KOH and sphingolipids were extracted with 1-butanol (containing 17:0 ceramide (C17:0 Cer) and C17 dihydrosphingosine (C17 dhSph) as internal standards). Vacuum-dried lipid extracts were resuspended in 100 µL acetonitrile/methanol/water (47.5:47.5:5 (v:v:v), 0.1% formic acid) and deuterium label bearing, *de novo*-formed sphingolipid species (3KDS-d_5, dhSph-d_5 and C16:0 dhCer-d_8) were analyzed by HPLC-MS/MS as described (2).

Determination of intracellular Des activity by HPLC-MS/MS

Primary human monocytes were differentiated in the presence of FOH (50 µM and 100 µM), methanol or left untreated for 24 h [with and without *N*-acetyl-L-cysteine (NAC) (Sigma-Aldrich)], 72 h, and 144 h, as described above. Some cells were treated with GW9662 prior to FOH treatment or with RSG for 24 h and 72 h. Suspensions of cells were incubated for 4 h

with 1 µM of the stable isotope-labeled Des substrate d_7-C13:0 dhCer (Avanti Polar Lipids) and were subsequently subjected to lipid extraction. The extraction solvent, however, solely contained C17:0 Cer as internal standard. A 1290 Infinity II HPLC coupled to a 6465B (Ultivo) triple-quadrupole mass spectrometer (both Agilent Technologies) was used for analysis, and instrumental parameters were maintained (if possible) compared with the sphingolipid profiling described above. The electrospray ion source was operated in ESI+ mode and the following MRM transitions were recorded to monitor cellular Des activity (collision energies are given in parentheses): *m/z* 505.5 → 487.5 (16 eV, quantifier), *m/z* 505.5 → 291.3 (28 eV), and *m/z* 505.5 → 273.3 (32 eV) for d_7-C13:0 dhCer; *m/z* 485.5 → 289.3 (20 eV), *m/z* 485.5 → 271.3 (20 eV, quantifier), and *m/z* 485.5 → 259.2 (20 eV) for d_7-C13:0 Cer. To this end, peak areas of the substrate d_7-C13:0 dhCer (eluting at 12.3 min) as well as the product d_7-C13:0 Cer (eluting at 11.9 min) were normalized to the internal standard C17:0 Cer and quantified via C16:0 Cer external calibration using MassHunter Quantitative Analysis software (version 10.1, Agilent Technologies). Analytical details for analysis of C16:0 Cer and C17:0 Cer can be taken from Suppl. Table 1. Intracellular Des activity was determined as conversion rate (%) = c (d_7-C13:0 Cer) / (c (d_7-C13:0 dhCer) + c (d_7-C13:0 Cer)) with c being the concentration (in nmol/L) in the lipid extract.

Mitochondrial respiration

Untreated DCs or those differentiated in the presence of FOH (50 µM and 100 µM), with and without pre-treatment of PPAR-γ antagonist GW9662 (10 µM) or solvent as control, were collected after 24 h, 72 h, and 144 h, washed twice with phosphate buffered saline (PBS) and resuspended in Seahorse XF RPMI Medium (pH 7.4) supplemented with pyruvate (1 mM), L-glutamine (2 mM) and 10 mM glucose (all Agilent Technologies). Cells were seeded at 10^5 cells/well (180 µL) on poly-D-lysin-coated Seahorse XFe96/XF Pro cell culture microplates, then incubated for 1 h at 37°C in a non-CO_2 incubator prior to the start of the assay. Seahorse XFe96/XF Pro Sensor Cartridges were hydrated with Seahorse XF Calibrant Solution (Agilent Technologies) overnight at 37°C in a non-CO_2 incubator. The Mito Stress Test Assay was performed on an Extracellular Flux Analyzer, Seahorse XFe96 Pro Analyzer (Agilent

Technologies) to measure oxygen consumption rate (OCR) and extracellular acidification rate (ECAR). First, basal OCR was measured, followed by proton leak detection through ATP synthesis inhibition using oligomycin complex (2 µM; Cayman Chemical). Maximal respiration was induced using FCCP (carbonyl cyanide 4-(trifluoromethoxy)phenylhydrazone; 1 µM; Cayman Chemical). Lastly, mitochondrial oxygen consumption was inhibited by rotenone (0.5 µM; AdipoGen Life Sciences) and antimycin A (0.5 µM; Sigma-Aldrich). The spare respiratory capacity represents the difference between maximal and basal respiration. ATP-linked respiration shows the difference between basal respiration and oligomycin-induced ATP synthesis-inhibited respiration.

RNA isolation and quantitative real-time PCR

Whole cell RNA was isolated using the RNeasy Plus Mini Kit (Qiagen) according to manufacturer's instructions. cDNA was generated using the LunaScript RT SuperMix Kit (New England Biolabs). For qPCR, the Luna Universal qPCR Master Mix (New England Biolabs) was added to 50 ng cDNA to detect *DEGS1* gene expression (Qiagen, QuantiTect Primer Assay) using a qTOWER^{3}G Thermocycler (Analytik Jena). Gene expression was normalized to the beta-actin reference gene and $2^{-\Delta\Delta CT}$ values were calculated relative to the untreated control.

ATP production in monocyte-derived dendritic cells

ATP production in DCs differentiated in the presence of FOH (50 µM and 100 µM) was determined using CellTiter-Glo 2.0 Viability/ATP Assay from Promega according to the manufacturer's instructions. Amount of ATP detected was calculated by ATP standard curve.

Mitochondrial membrane potential

Mitochondrial membrane potential (Δψm) was detected during DC differentiation (after 24 h and 144 h). Cells were stained with 1 µM rhodamine 123 (Sigma-Aldrich) for 20 min at 37°C with 5 % CO_2 and analyzed by flow cytometry.

Mitochondrial mass

Mitochondrial mass was analyzed in immature DCs after 144 h. DCs were different in the presence of FOH (50 µM and 100 µM), GW9662 (10 µM) with and without addition of FOH, solvent control or left untreated. After 6 days, DCs were stained with 100 nM MitoTracker™ Green FM (Invitrogen) for 30 min at 37°C with 5 % CO_2 and analyzed by flow cytometry.

Viability

Viability in DCs (144 h) was determined using Annexin V-FITC Kit and propidium iodide (Miltenyi Biotec) according to the manufacturer's instructions.

Autophagy

Autophagy was determined by intracellular LC3 stain. Immature DCs (144 h) were fixed, permeabilized and stained with Anti-LC3 antibody (rabbit, Sigma-Aldrich) for 1 h at 4°C and secondarily labelled with AF488 antibody. LC3 fluorescence intensity was determined by flow cytometry.

Phagocytosis of *Candida albicans*

Phagocytosis of *C. albicans* (SC5314) was measured using DCs differentiated with and without FOH (50 µM and 100 µM) or solvent control. A BFP-expressing *C. albicans* strain SC5314 (*ADH1/adh1::BFP-SAT1*) (3) was cultured in liquid YPD medium at 30°C overnight. Germ tube formation was induced using RPMI 1640 medium for 1 h at 37°C. *C. albicans* was confronted with DCs (MOI = 1) in RPMI 1640 medium supplemented with 5% FBS for 1 h at 37°C or 4°C for the control. Following confrontation, cells were washed with PBS and stained with CD11c-APC antibody (Miltenyi Biotec) to detect DCs and FITC anti-*Candida albicans* antibody (OriGene) to stain extracellular *Candida*. Phagocytic activity was determined using flow cytometry and normalized to the untreated control.

Thimerosal-killed *Candida albicans*

C. albicans strain SC5314 was cultivated on yeast extract peptone dextrose (YPD) agar plates. The yeast cells were then grown in 5 mL liquid YPD medium at 30°C with shaking at 180 rpm overnight. After 3 washing steps using HBSS buffer at 12000 rpm, *Candida* cells were killed by incubation in 0.5% Thimerosal (Sigma-Aldrich) for 1 h at 30°C with shaking at 180 rpm. Control aliquots were plated on YPD agar plates and incubated at 37°C for up to 2 days to confirm non-viability.

***Candida albicans* biofilm**

C. albicans strains (SC5314 and ATCC10231) were grown in liquid YPD at 30°C with shaking at 180 rpm overnight. After cell counts were performed, 10^5 *Candida* cells were plated in 6-well plates (3 mL liquid YPD medium per well) and incubated at 37°C with 5 % CO_2 overnight. Next, the biofilm was washed three times with HBSS buffer and 3 mL RPMI 1640 medium supplemented with 10% heat inactivated FBS, 10 mM L-glutamine, 100 U/mL penicillin-streptomycin, IL-4 (1000 U/mL) and GM-CSF (800 U/mL) was added (per well). Transwell inserts containing $2*10^6$ freshly isolated primary human $CD14^+$ monocytes were carefully placed on top of the *C. albicans* biofilms. Monocytes were differentiated over 6 days with a medium exchange and re-supplementation of IL-4 and GM-CSF on day 3.

SUPPLEMENTARY TABLES AND FIGURES

Group	Compound	Precursor ion (*m/z*)	Product ion (*m/z*) [a]	Retention time (min)	ISTD
**LCBs**	Sph	300.3	**282.3 (8)** / 252.3 (16)	5.5	d_7-Sph
	dhSph	302.3	**284.3 (12)** / 254.3 (20)	5.8	d_7-dhSph
	S1P	380.3	**264.3 (20)** / 82.1 (32)	6.8	d_7-S1P
	dhS1P	382.3	**284.3 (12)** / 266.4 (16)	7.2	d_7-S1P
**Cer**	C16:0 Cer	520.5	**264.3 (24)** / 282.3 (24)	13.7	C17:0 Cer
	C18:0 Cer	548.5	**264.2 (24)** / 282.3 (28)	15.6	C17:0 Cer
	C20:0 Cer	576.6	**264.3 (32)** / 282.3 (28)	18.0	C17:0 Cer
	C22:0 Cer	604.6	**264.3 (34)** / 282.3 (30)	21.0	C17:0 Cer
	C24:0 Cer	632.6	**264.3 (36)** / 282.3 (28)	24.5	C17:0 Cer
	C24:1 Cer	630.6	**264.3 (36)** / 282.3 (32)	21.2	C17:0 Cer
**dhCer**	C16:0 dhCer	540.5	**522.6 (20)** / 284.3 (28)	14.2	C17:0 Cer
	C18:0 dhCer	568.5	**550.5 (20)** / 284.3 (28)	16.3	C17:0 Cer
	C20:0 dhCer	596.6	**578.6 (22)** / 284.3 (32)	18.9	C17:0 Cer
	C22:0 dhCer	624.6	**606.6 (22)** / 284.3 (32)	22.1	C17:0 Cer
	C24:0 dhCer	652.7	**634.6 (24)** / 284.3 (36)	25.7	C17:0 Cer
	C24:1 dhCer	650.7	**632.7 (24)** / 284.3 (36)	22.3	C17:0 Cer
**SM**	C16:0 SM	703.6	**184.0 (8)** / 86.1 (76)	12.8	d_{31}-C16:0 SM
	C18:0 SM	731.6	**184.0 (28)** / 86.1 (76)	14.7	d_{31}-C16:0 SM
	C20:0 SM	759.6	**184.0 (28)** / 86.1 (78)	17.0	d_{31}-C16:0 SM
	C22:0 SM	787.7	**184.0 (28)** / 86.1 (78)	19.4	d_{31}-C16:0 SM
	C24:0 SM	815.7	**184.0 (28)** / 86.1 (80)	22.7	d_{31}-C16:0 SM
	C24:1 SM	813.7	184.0 (8) / **86.1 (80)**	19.5	d_{31}-C16:0 SM
**dhSM**	C16:0 dhSM	705.6	**184.0 (8)** / 86.1 (76)	13.5	d_{31}-C16:0 SM
	C18:0 dhSM	733.6	**184.0 (28)** / 86.1 (76)	15.6	d_{31}-C16:0 SM
	C20:0 dhSM	761.6	**184.0 (28)** / 86.1 (78)	18.0	d_{31}-C16:0 SM
	C22:0 dhSM	789.7	**184.0 (28)** / 86.1 (78)	20.9	d_{31}-C16:0 SM
	C24:0 dhSM	817.7	**184.0 (28)** / 86.1 (80)	24.5	d_{31}-C16:0 SM
	C24:1 dhSM	815.7	184.0 (8) **/ 86.1 (80)**	20.9	d_{31}-C16:0 SM
**1-deoxy-SL**	1-deoxy-dhSph	286.3	**268.3 (16)** / 55.2 (36)	6.0	C17:0 Cer
	C16:0 1-deoxy-dhCer	524.5	**268.3 (32)** / 506.6 (20)	15.3	C17:0 Cer
	C24:1 1-deoxy-dhCer	634.7	**268.3 (36)** / 616.7 (24)	24.3	C17:0 Cer
	C16:0 1-deoxy-Cer	504.5	**266.3 (24)** / 70.2 (48)	14.8	C17:0 Cer
	C24:1 1-deoxy-Cer	614.6	**266.3 (28)** / 70.1 (68)	23.4	C17:0 Cer
**ISTD**	d_7-Sph	307.3	**289.3 (8)** / 259.3 (20)	5.5	-
	d_7-dhSph	309.4	**291.3 (12)** / 261.3 (24)	5.8	-
	d_7-S1P	387.3	**271.3 (20)** / 82.1 (36)	6.8	-
	C17:0 Cer	534.5	**264.3 (24)** / 282.3 (28)	14.6	-
	d_{31}-C16:0 SM	734.6	**184.0 (28)** / 86.1 (76)	12.7	-

Table S1: HPLC-MS/MS parameter for quantification of cellular sphingolipids (SL).

[a] Quantifier mass transitions are shown in bold. Collision energies (in eV) are shown in parentheses.

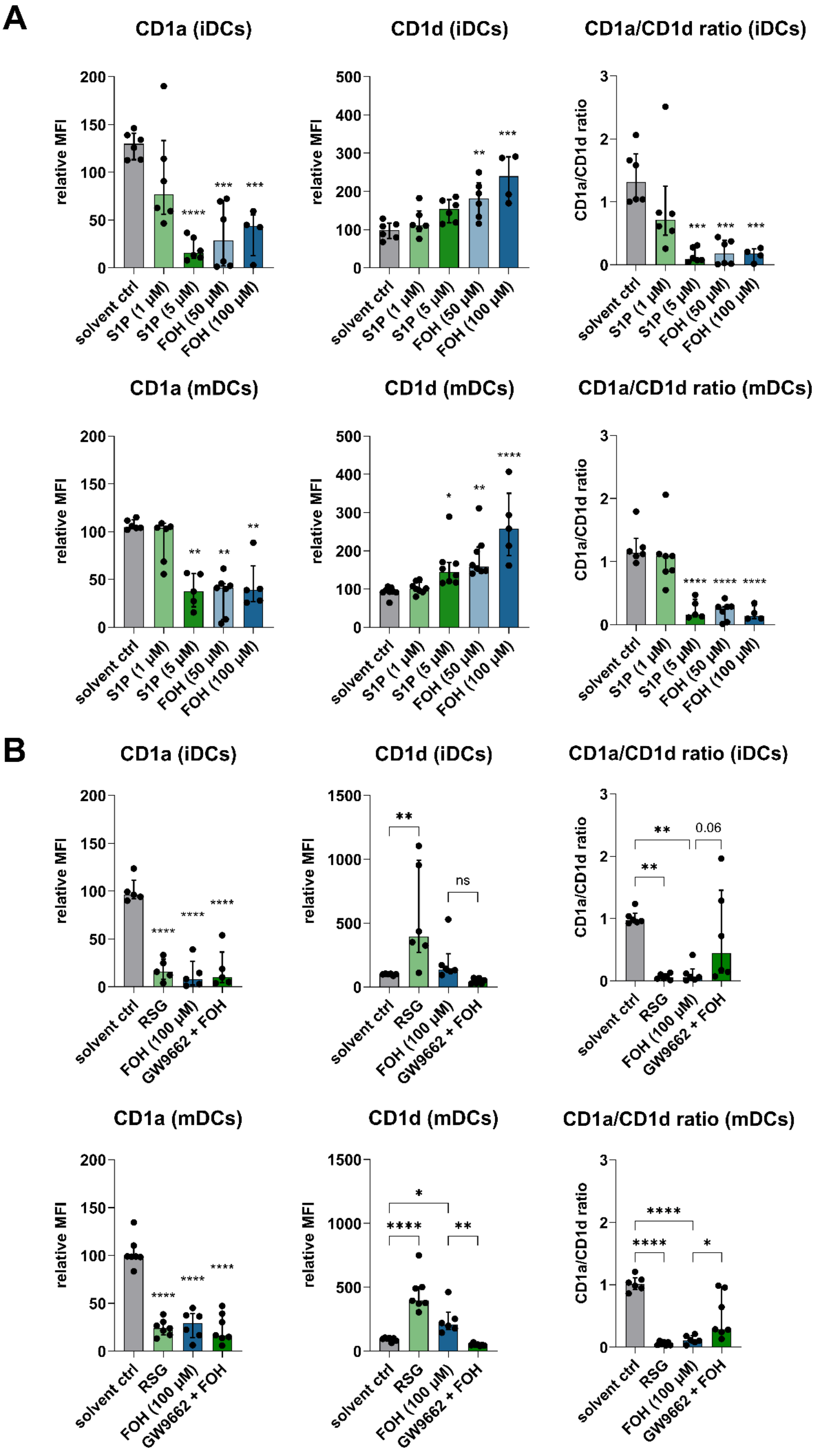
A
CD1a (iDCs)
CD1d (iDCs)
CD1a/CD1d ratio (iDCs)
CD1a (mDCs)
CD1d (mDCs)
CD1a/CD1d ratio (mDCs)
relative MFI
CD1a/CD1d ratio
solvent ctrl
S1P (1 µM)
S1P (5 µM)
FOH (50 µM)
FOH (100 µM)
B
CD1a (iDCs)
CD1d (iDCs)
CD1a/CD1d ratio (iDCs)
CD1a (mDCs)
CD1d (mDCs)
CD1a/CD1d ratio (mDCs)
solvent ctrl
RSG
FOH (100 µM)
GW9662 + FOH
ns
0.06

Figure S1. CD1a and CD1d expression of immature DCs (iDCs) and mature DCs (mDCs). (**A**) iDCs were differentiated in the presence of sphingosine 1-phosphate (S1P; 1 µM and 5 µM), FOH (50 µM and 100 µM) or their solvent control for 6 days (144 h). (**B**) Additionally, iDCs were differentiated in the presence of the PPAR-γ agonist rosiglitazone (RSG; 5 µM) or the PPAR-γ antagonist GW9662 (10 µM) prior to FOH (100 µM) administration. iDCs were treated with LPS (100 ng/mL) for 24 h to induce maturation into mDCs. Data from six individual experiments were normalized to the MFI of untreated control cells (set to 100%, not shown in the bar chart) and groups were statistically compared to the solvent control group using one-way ANOVA with Dunnett's post-hoc test (ns, not significant; *, *P<0.05*; **, *P<0.01*; ***, *P<0.001*; ****, *P<0.0001*).

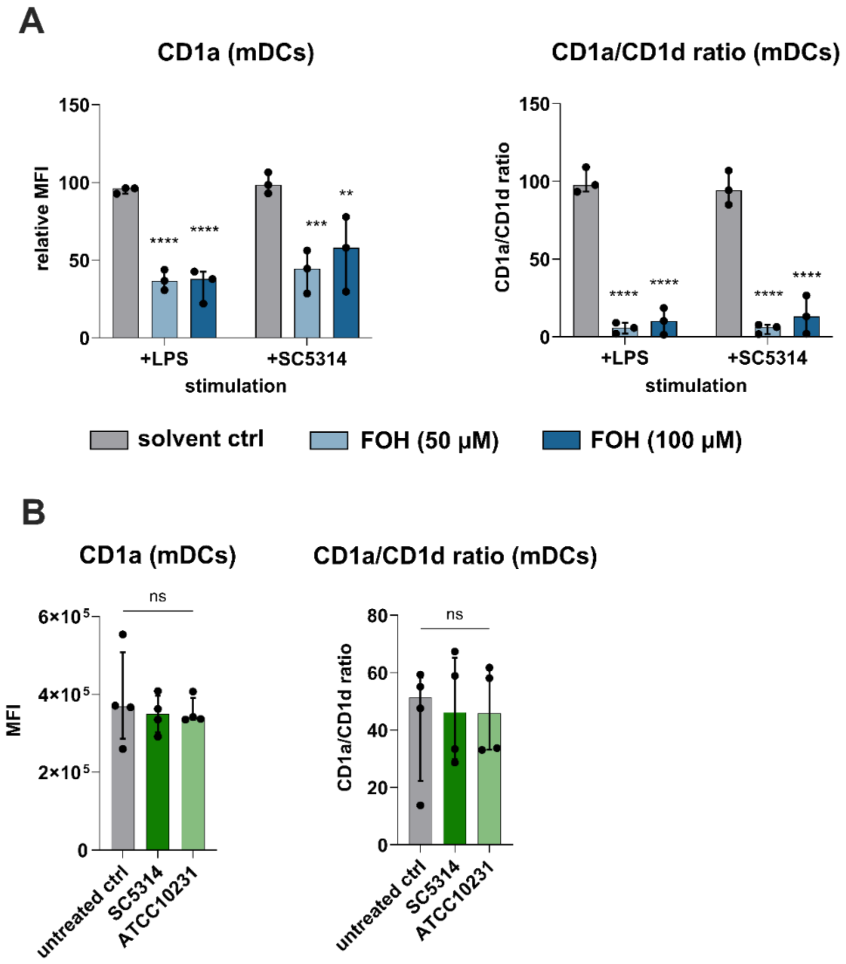

Figure S2. CD1a expression and CD1a/CD1d ratio in mature DCs (mDCs). (**A**) DCs were differentiated in the presence of FOH (50 µM and 100 µM) or solvent control for 6 days (144 h) and matured in the presence of LPS (100 ng/mL) or thimerosal-killed *C. albicans* SC5314 (MOI = 1) for 24 h. Data from three individual experiments were normalized to the MFI of untreated control cells (set to 100%, not shown in the bar chart) and groups were statistically compared to the solvent control group using one-way ANOVA with Dunnett's post-hoc test. (**B**) DCs differentiated in the presence of Thimerosal-killed *C. albicans* strains SC5314 and ATCC10231 (MOI = 0.5) and matured using LPS (100 ng/mL) for 24 h (n = 4). ns, not significant; **, *P<0.01*; ***, *P<0.001*; ****, *P<0.0001*.

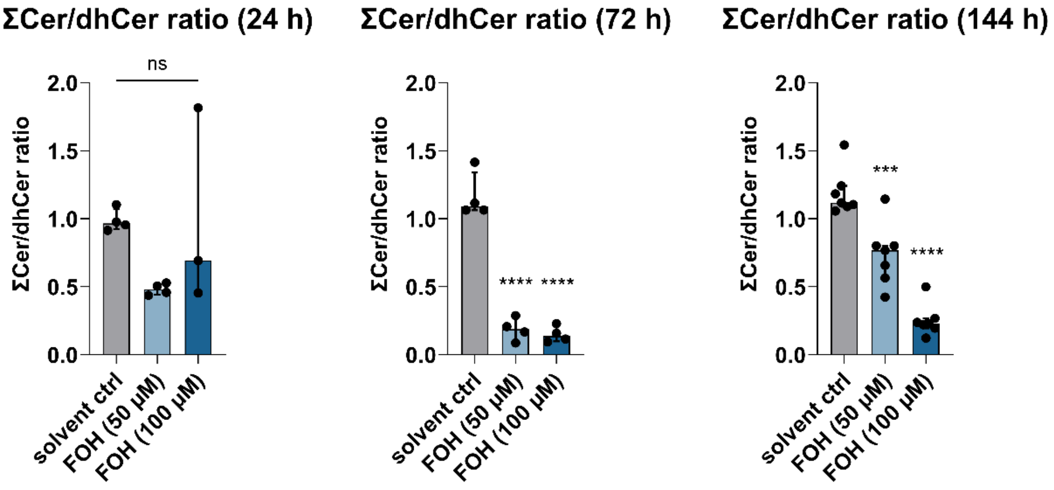

Figure S3. Total Cer to dhCer ratio in DCs differentiated from monocytes in the presence of FOH (50 µM and 100 µM) or solvent control (n = 4). Data were normalized to the Cer/dhCer ratio of untreated control cells (set to 100%, not shown in the bar chart) and statistical comparisons were conducted between the solvent control group and the FOH groups using one-way ANOVA with Dunnett's post-hoc test (ns, not significant; ***, *P<0.001*; ****, *P<0.0001*).

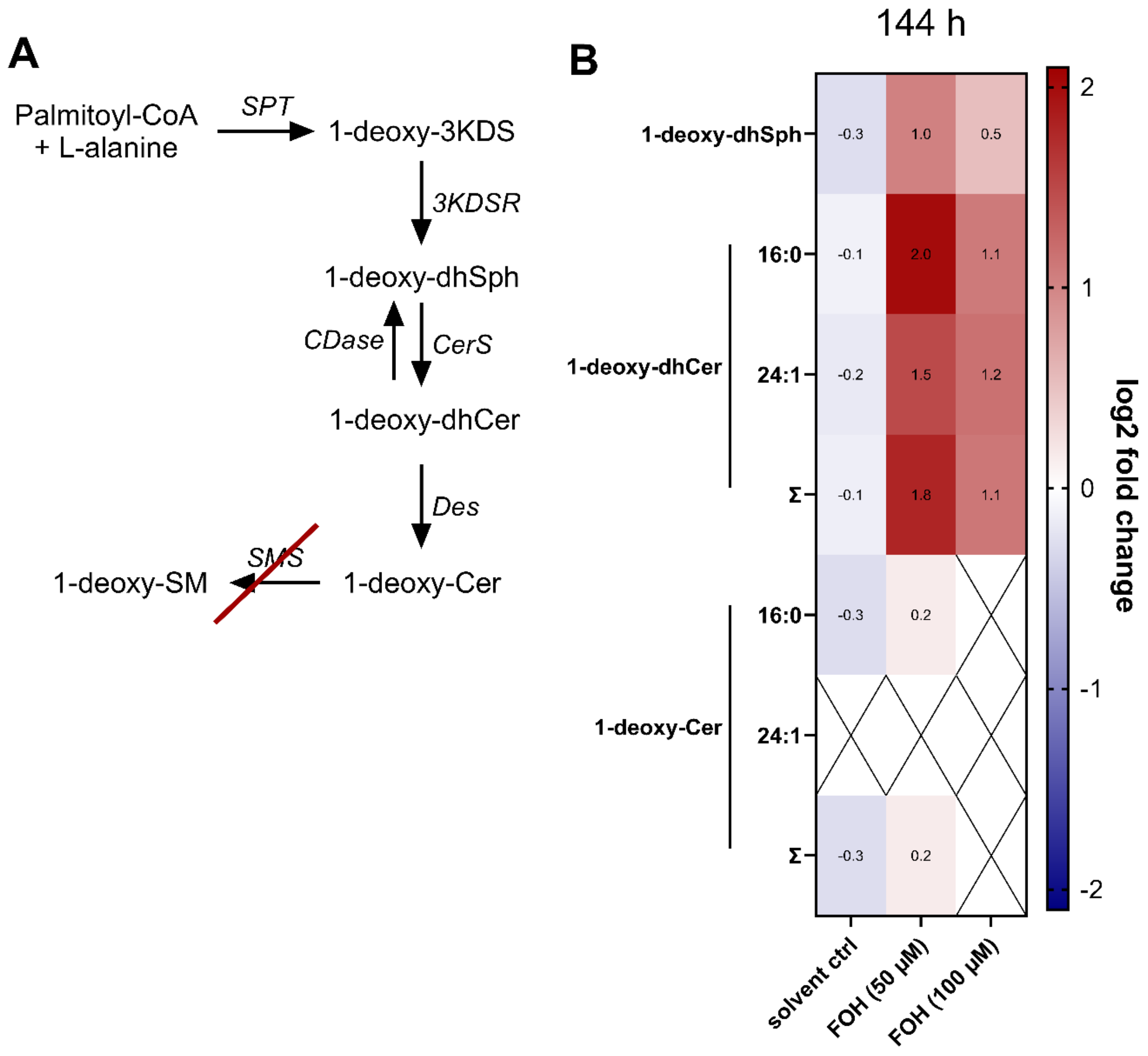

Figure S4. FOH affects 1-deoxysphingolipid synthesis in DCs. (**A**) In 1-deoxysphingolipid synthesis, L-alanine is used instead of L-serine for the condensation reaction catalyzed by SPT, resulting in failure in the downstream synthesis of more complex sphingolipids such as 1-deoxy-SM. (**B**) 1-deoxysphingolipid species were quantified by HPLC-MS/MS in DCs differentiated from monocytes (144 h) treated with FOH (50 µM and 100 µM) or solvent control (n = 4). Data were normalized to the untreated control group (not shown) and presented as log2 fold change. X, not detectable. SPT, serine palmitoyltransferase; 1-deoxy-3KDS, 1-deoxy-3-keto-dihydrosphingosine; 3KDSR, 3-keto-dihydrosphingosine reductase; 1-deoxy-dhSph, 1-deoxy-dihydrosphingosine; 1-deoxy-dhCer, 1-deoxy-dihydroceramide; 1-deoxy-Cer, 1-deoxy-ceramide; 1-deoxy-SM, 1-deoxy-sphingomyelin; CerS, ceramide synthase; CDase, ceramidase; Des, dihydroceramide desaturase; SMS, sphingomyelin synthase.

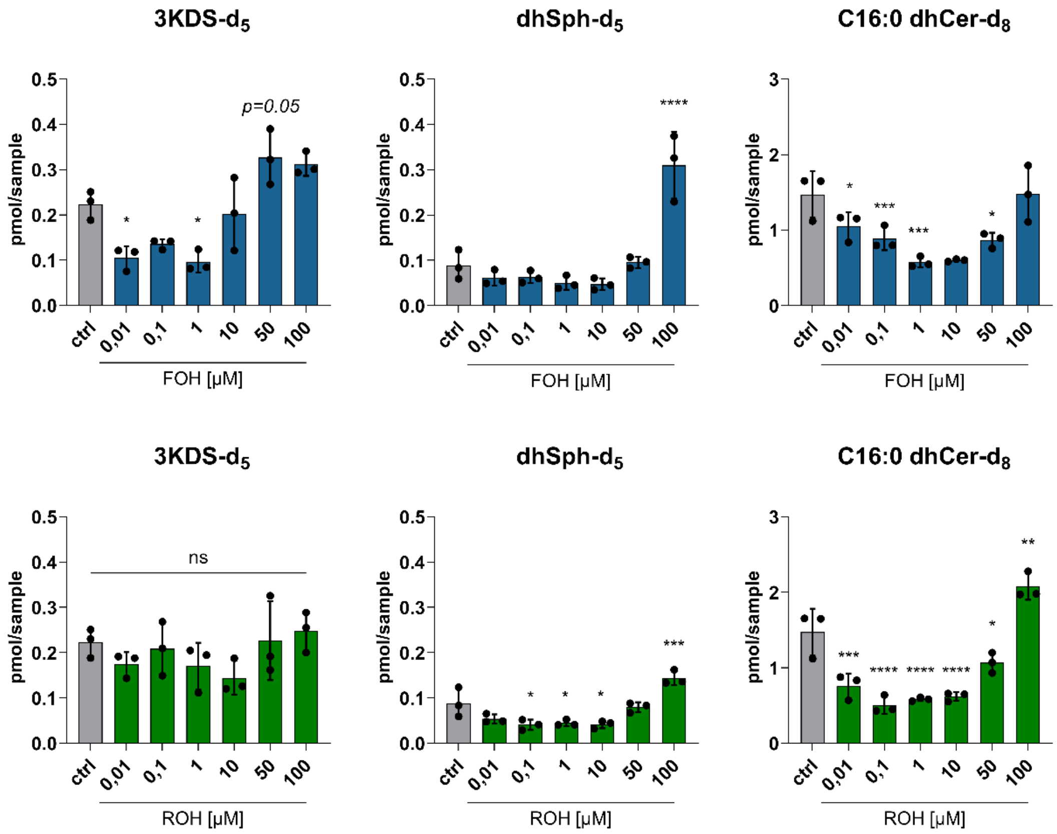

Figure S5. Dose-dependent effects (0.01 – 100 µM) of farnesol (FOH) and retinoic acid (ROH) on deuterium-labelled 3KDS-d_5, dhSph-d_5 and C16:0 dhCer-d_8 detection in a cell-free sphingolipid *de novo* synthesis assay (applied substrates: palmitate-d_3 and L-serine-d_3). Data from three independent experiments were used to calculate comparisons between FOH-treated samples or ROH-treated samples and the control group. Statistical analyses were performed using one-way ANOVA with Dunnett's multiple comparisons test (ns, not significant; *, *P<0.05*; **, *P<0.01*; ***, *P<0.001*; ****, *P<0.0001*). 3KDS, 3-ketodihydrosphingosine; dhSph, dihydrosphingosine; dhCer, dihydroceramide.

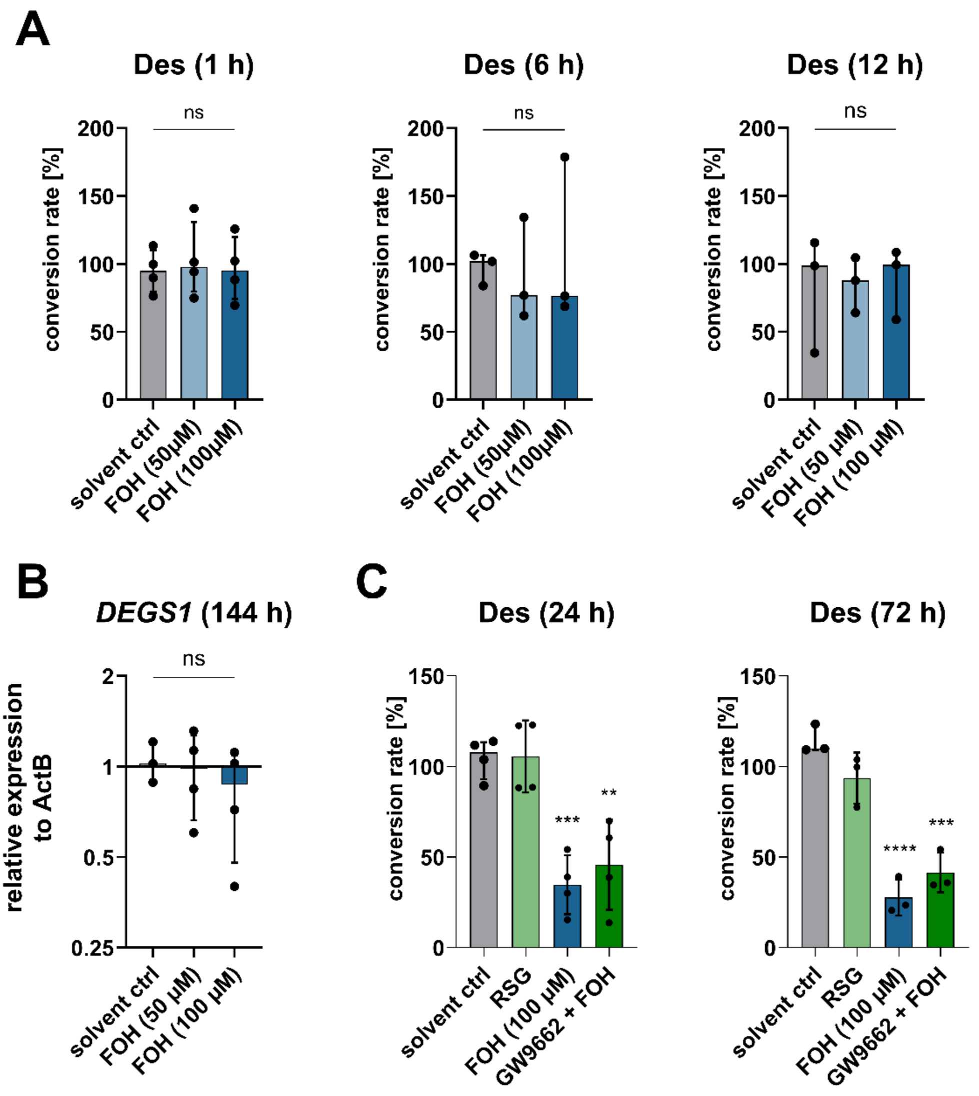

Figure S6. Des activity in monocytes differentiating into DCs at early time points. (**A**) Des activity was measured in cells treated with FOH (50 µM and 100 µM) or solvent control after 1 h (n = 4), 6 h (n = 3) and 12 h (n = 3). The d_7-C13:0 dhCer (substrate) and d_7-C13:0 Cer (product) content was measured using HPLC-MS/MS, and conversion rates were calculated and normalized to those of the untreated control group (set to 100%, not shown in the bar chart). (**B**) Gene expression of *DEGS1* relative to beta-actin (ActB) in DCs (144 h) differentiated in the presence of FOH (50 µM and 100 µM) or solvent control; normalized to the untreated control (not shown; n = 3). (**C**) Des activity in differentiating DCs treated with PPAR-γ agonist RSG (5 µM), FOH (100 µM), or PPAR-γ antagonist GW9662 (10 µM) prior to FOH (100 µM) stimulation after 24 h (n = 4) and 72 h (n = 3). ns, not significant; **, *P<0.01*; ***, *P<0.001*; ****, *P<0.0001*.

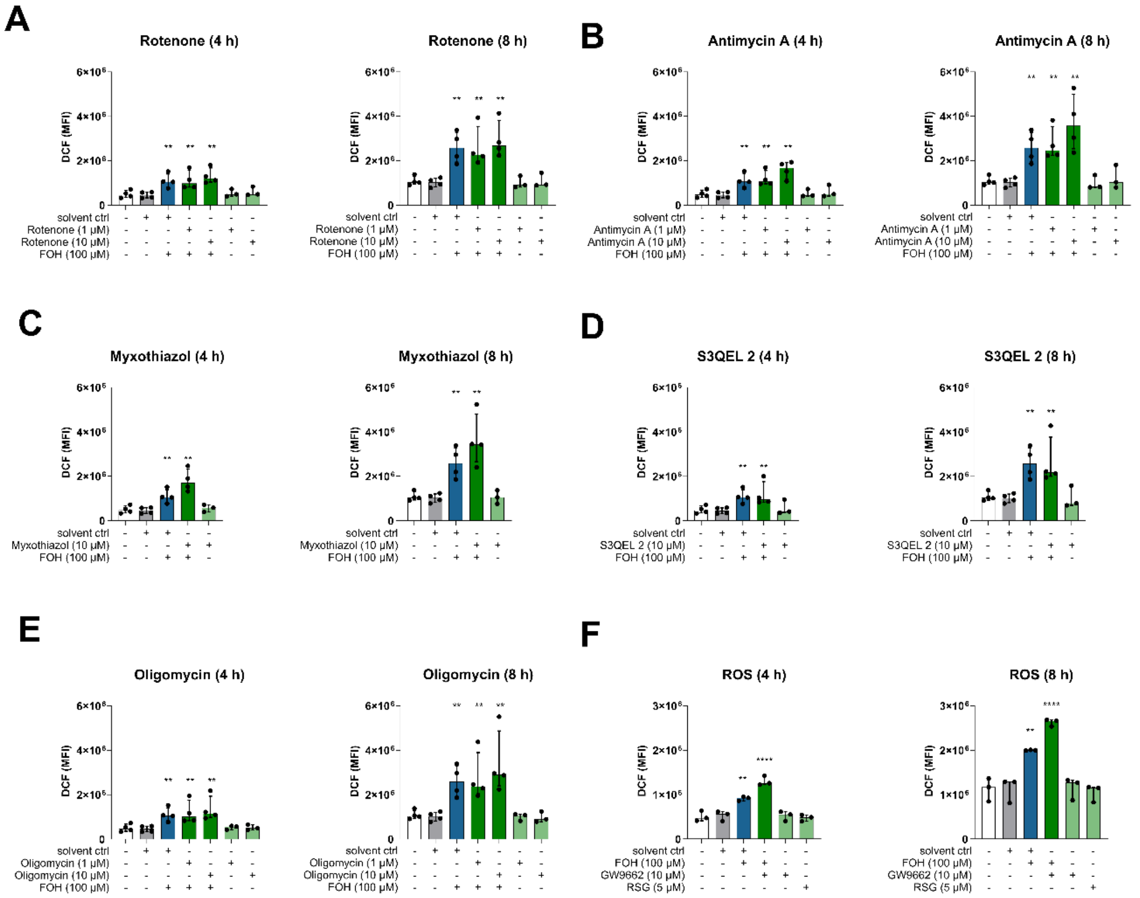

Figure S7. ROS generation was determined in monocytes differentiating into DCs in the presence of FOH (100 µM) or solvent control for 4 h. One hour prior to the addition of FOH, (**A**) rotenone (1 µM and 10 µM), (**B**) antimycin A (1 µM and 10 µM), (**C**) myxothiazol (10 µM), (**D**) S3QEL 2 (10 µM), (**E**) oligomycin (1 µM and 10 µM) or (**F**) GW9662 (10 µM) were added (n = 4). Cellular ROS production was determined using the DCF assay, (**F**) also in RSG (5 µM)-treated cells. Significance was determined using one-way ANOVA with Dunnett's post-hoc test (ns, not significant; **, *P<0.01*; ****, *P<0.0001*).

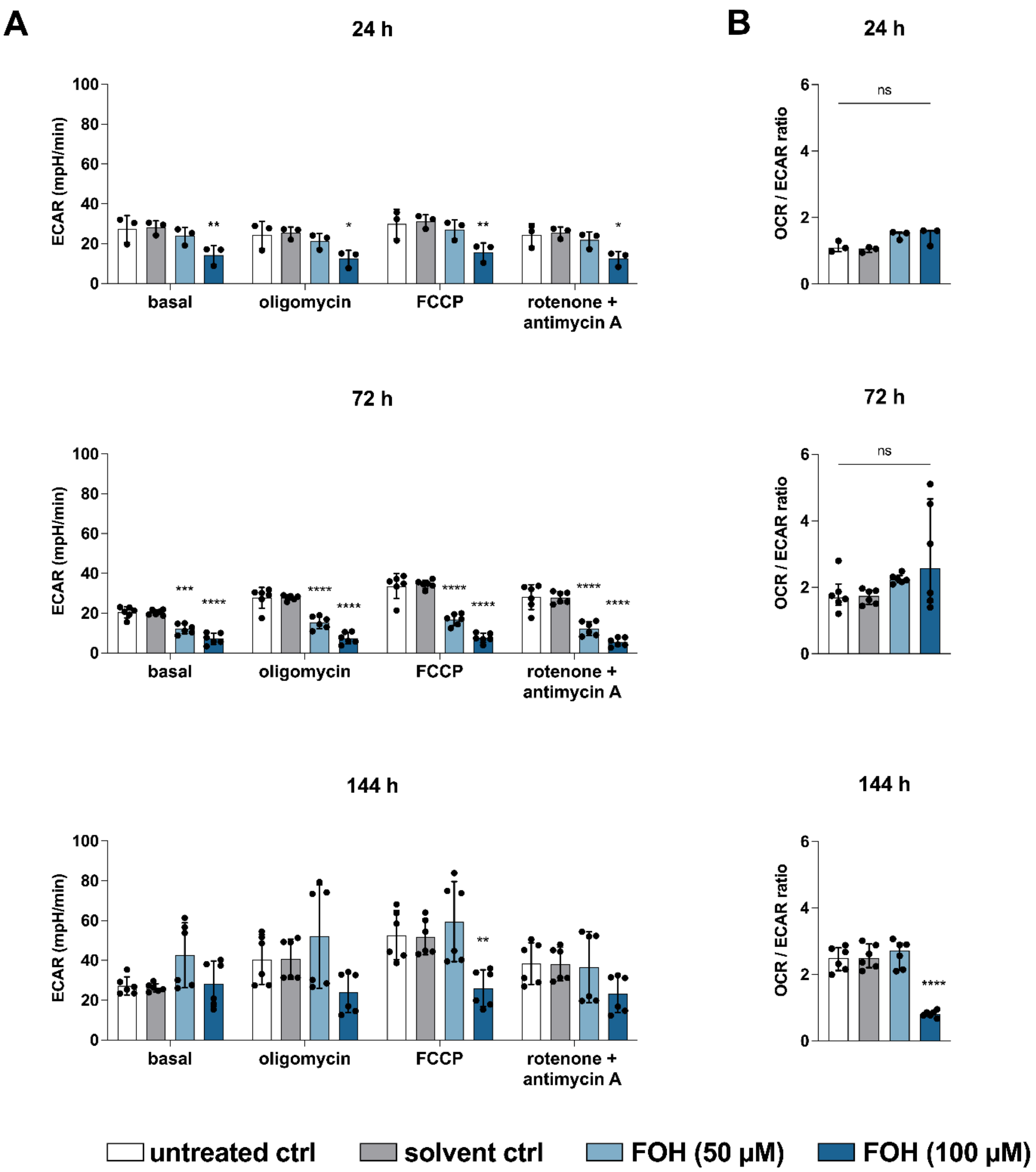

Figure S8. The Seahorse XF Cell Mito Stress Test was performed to evaluate mitochondrial respiration by measuring the oxygen consumption rate (OCR) and the extracellular acidification rate (ECAR). (**A**) ECAR values of DCs differentiated over 24 h (n = 3), 72 h (n = 6) and 144 h (n = 6) in the presence of FOH (50 µM or 100 µM), solvent control or left untreated. (**B**) Basal OCR to basal ECAR ratio during DC differentiation (24 h, 72 h, 144 h). ns, not significant; *, *P<0.05*; **, *P<0.01*; ***, *P<0.001*; ****, *P<0.0001*.

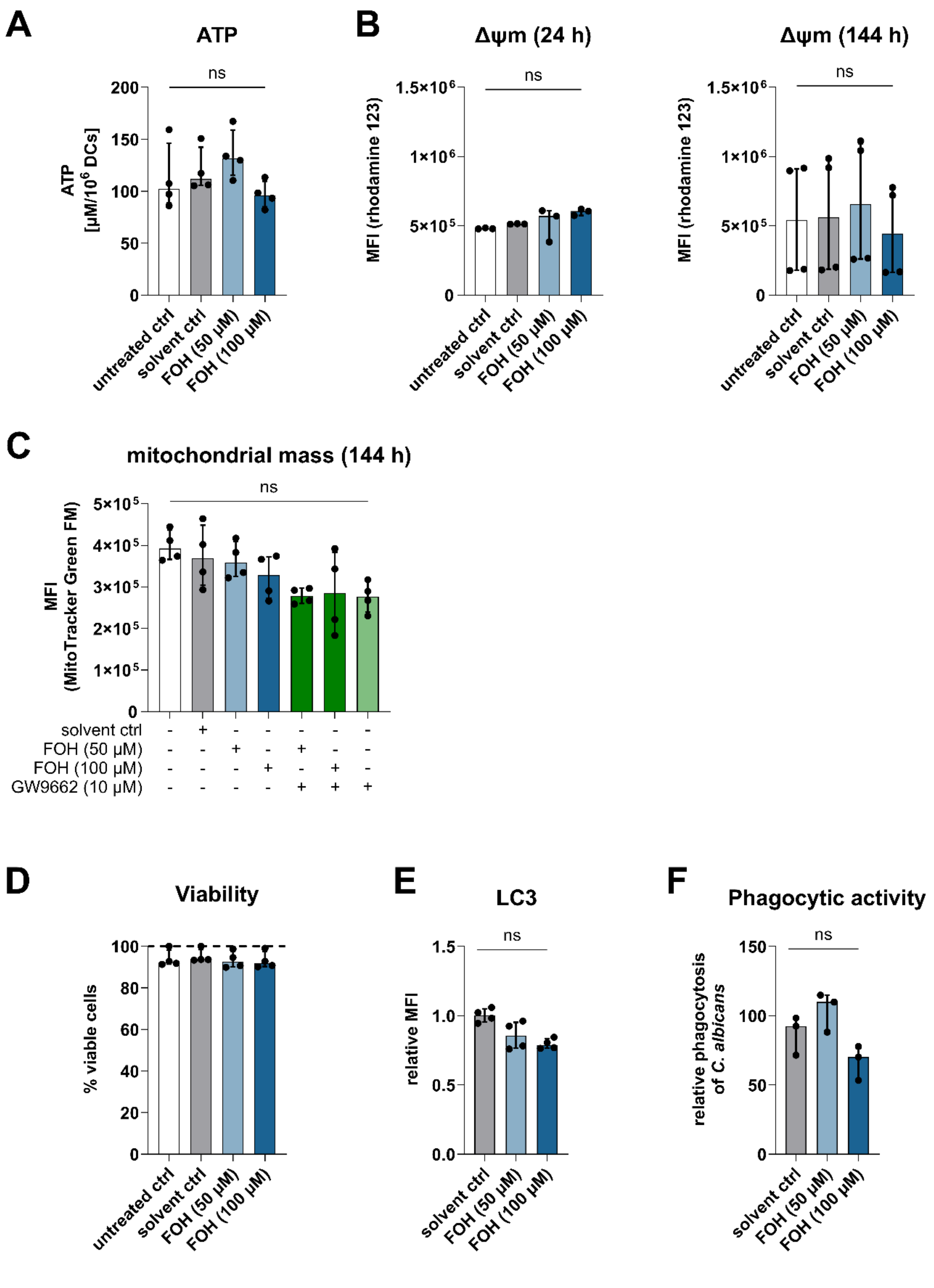

Figure S9. (**A**) ATP production in immature DCs (144 h) differentiated in the presence of FOH (50 µM and 100 µM), methanol as a solvent control or left untreated (n = 4). (**B**) Mitochondrial membrane potential (Δψm) detected using rhodamine 123 by flow cytometry in untreated DCs and those treated with FOH (50 µM and 100 µM) or solvent control for 24 h (n = 3) and 144 h (n = 4). (**C**) Mitochondrial mass detected using MitoTracker Green FM by flow cytometry in untreated DCs and those differentiated for 144 h in the presence of FOH, GW9662 (10 µM) or solvent control (n = 4). (**D**) Viability in immature DCs (144 h) determined by flow cytometry using Annexin V and PI staining. (**E**) Intracellular LC3 staining in DCs differentiated with solvent

control or FOH (50 µM and 100 µM) for 144 h (n = 4). LC3 staining intensity was determined by flow cytometry and normalized to the MFI of untreated control cells (set to 1, not shown in the bar diagram). (**F**) Phagocytic activity of DCs differentiated from monocytes in the presence of FOH (50 µM and 100 µM) or methanol as a solvent control (144 h). DCs were confronted with BFP-expressing *C. albicans* and phagocytic activity was determined by flow cytometry. An anti-*C. albicans* antibody was used to stain extracellular *Candida*. Data were normalized to untreated control DCs (set to 100%, not shown in the bar chart; n = 3). Statistical significance was calculated by one-way ANOVA using Tukey's post-hoc test. ns, not significant.

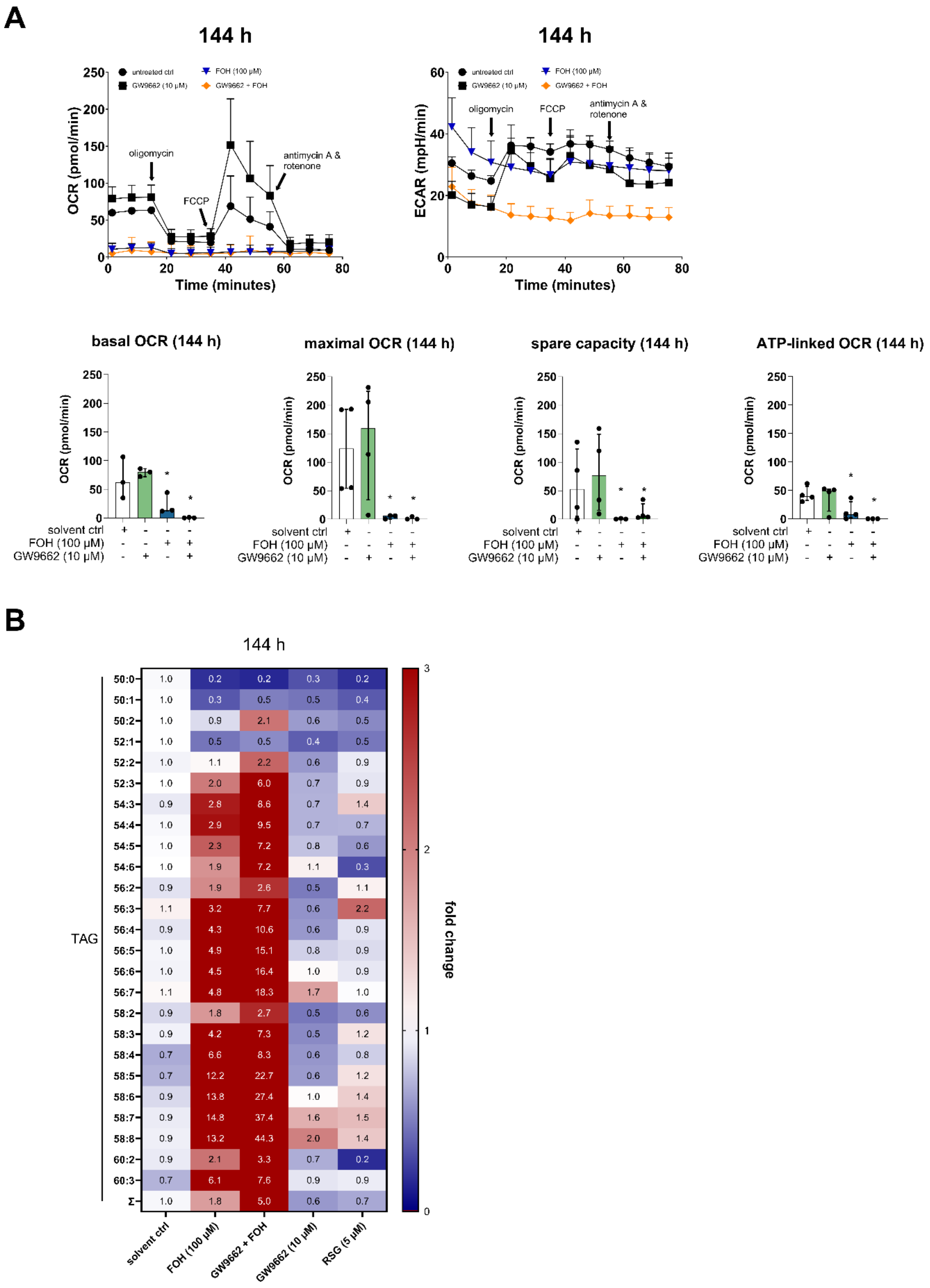

Figure S10. (**A**) Monocytes were stimulated with GM-CSF and IL-4 to induce differentiation into DCs in the presence or absence of FOH (100 µM), PPAR-γ antagonist GW9662 (10 µM) with and without FOH, or solvent control for 144 h (n = 4). The Seahorse XF Cell Mito Stress assay was performed to measure the mitochondrial OCR and ECAR and asses the role of PPAR-γ in FOH-induced changes in mitochondrial respiration. (**B**) Changes in triacylglycerol

(TAG) synthesis upon stimulation of monocytes with FOH (100 µM), GW9662 (10 µM) with and without FOH, RSG (5 µM) and solvent control for 144 h (n = 4). Fold change was calculated relative to the untreated control group (set to 1; not shown). Statistical comparison between groups was performed by one-way ANOVA with Tukey's multiple comparisons. ns, not significant; *, *P<0.01*.

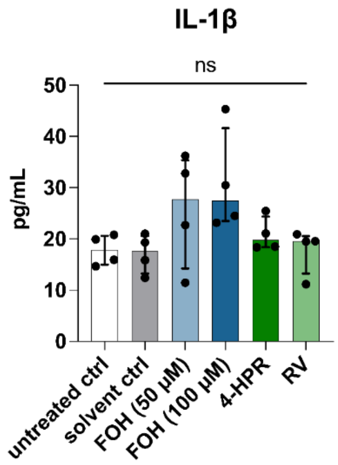

Figure S11. IL-1β secretion by DCs differentiated in the presence of solvent (control), FOH (50 µM and 100 µM), 4-HPR (5 µM), or RV (50 µM) after maturation with LPS (100 ng/mL) for 24 h (n = 4). ns, not significant.

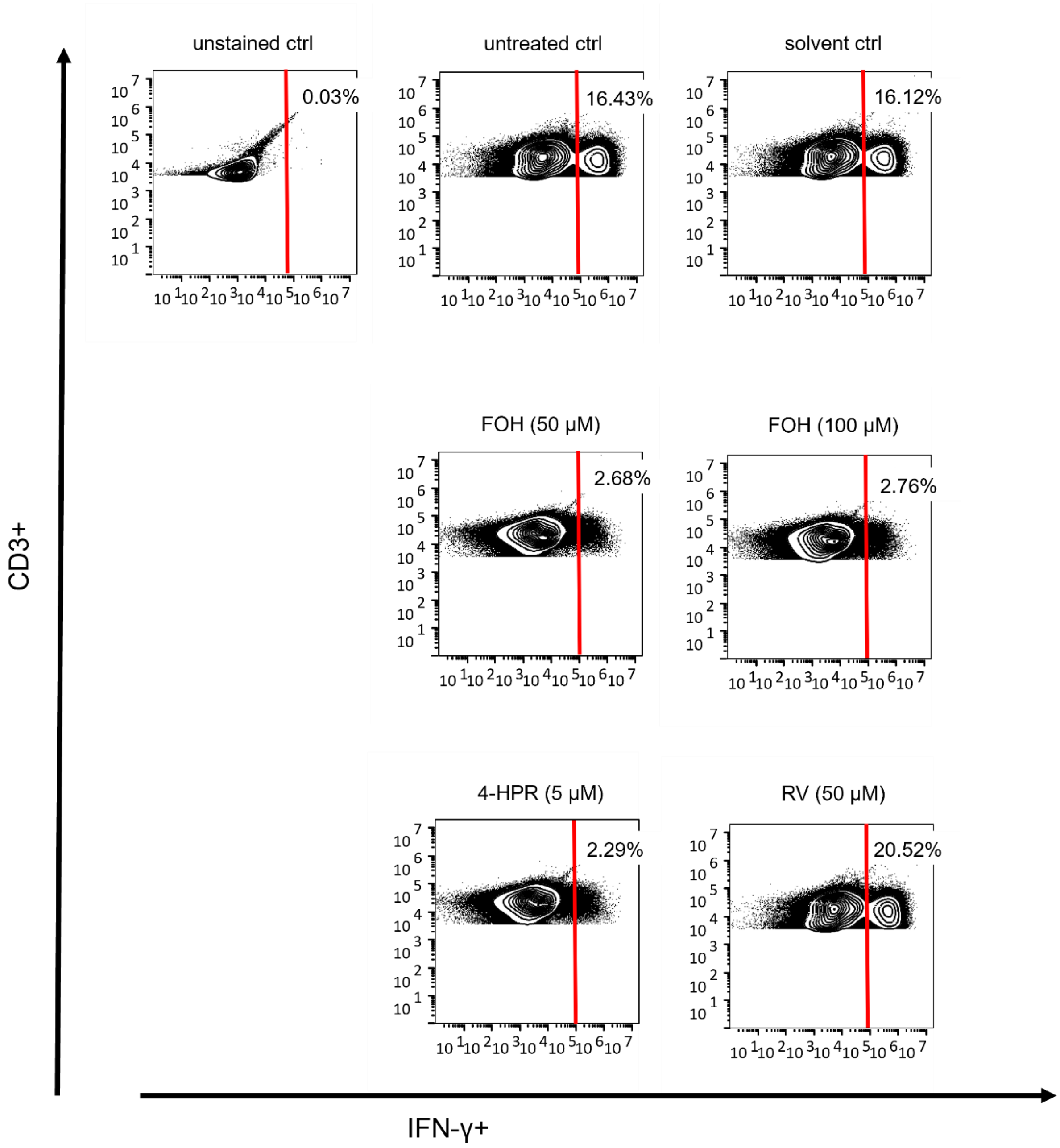

Figure S12. Representative flow cytometry plots of intracellular IFN-γ-staining in T cells. T cells were isolated from PBMCs and cultured at a 1:1 ratio with mature DCs. After 4 days, T cells were stimulated with PMA, ionomycin and brefeldin A for 4 h. Extracellular CD3 staining and intracellular IFN-γ staining were performed, and cells were analyzed by flow cytometry. Graphs were generated in FlowLogic 8.

SUPPLEMENTARY REFERENCES

1. Vivas W, Leonhardt I, Hünniger K, Häder A, Marolda A, Kurzai O. 2019. Multiple Signaling Pathways Involved in Human Dendritic Cell Maturation Are Affected by the Fungal Quorum-Sensing Molecule Farnesol. J Immunol 203:2959-2969.
2. Wigger D, Gulbins E, Kleuser B, Schumacher F. 2019. Monitoring the Sphingolipid de novo Synthesis by Stable-Isotope Labeling and Liquid Chromatography-Mass Spectrometry. Front Cell Dev Biol 7:210.
3. Duggan S, Essig F, Hünniger K, Mokhtari Z, Bauer L, Lehnert T, Brandes S, Häder A, Jacobsen ID, Martin R, Figge MT, Kurzai O. 2015. Neutrophil activation by Candida glabrata but not Candida albicans promotes fungal uptake by monocytes. Cell Microbiol 17:1259-76.
